# Recurrent or Refractory High-Grade Gliomas Treated by Convection-Enhanced Delivery of a TGFβ*2*-Targeting RNA Therapeutic: A Post-Hoc Analysis with Long-Term Follow-Up

**DOI:** 10.3390/cancers11121892

**Published:** 2019-11-28

**Authors:** Fatih M. Uckun, Sanjive Qazi, Larn Hwang, Vuong N. Trieu

**Affiliations:** 1 Immuno-Oncology Program, Oncotelic Inc., Agoura Hills, CA 91301, USA; sqazi@gustavus.edu (S.Q.); larn.hwang@oncotelic.com (L.H.); vtrieu@oncotelic.com (V.N.T.); 2 Department of Immuno-Oncology, Ares Pharmaceuticals, St. Paul, MN 55110, USA; 3 Department of Biology, Bioinformatics Program, Gustavus Adolphus College, St. Peter, MN 56082, USA

**Keywords:** RNA therapeutic, glioma, immuno-oncology

## Abstract

*Background*. OT101 is a first-in-class RNA therapeutic designed to abrogate the immunosuppressive actions of transforming growth factor beta 2 (TGFβ2). Here, we report our post-hoc analysis of the single-agent activity of OT101 in adult patients with recurrent and/or refractory (*R/R*) high-grade gliomas. *Methods*. In a Phase 2 clinical trial (ClinicalTrials.gov, NCT00431561), OT101 was administered to 89 *R/R* high-grade glioma (HGG) (anaplastic astrocytoma/AA: 27; glioblastoma multiforme/GBM: 62) patients with an intratumoral catheter using a convection enhanced delivery (CED) system. Seventy-seven patients (efficacy population; GBM: 51; AA: 26) received at least the intended minimum number of four OT101 treatment cycles. Response determinations were based on central review of magnetic resonance imaging (MRI) scans according to the McDonald criteria. Standard statistical methods were applied for the analysis of data. *Findings*. Nineteen patients had a complete response (CR) or partial response (PR) following a slow but robust size reduction of their target lesions (median time for 90% reduction of the baseline tumor volume = 11.7 months, range: 4.9–57.7 months). The mean log reduction of the tumor volume was 2.2 ± 0.4 (median = 1.4: range: 0.4–4.5) logs. In addition, seven patients had a stable disease (SD) lasting ≥6 months. For the combined group of 26 AA/GBM patients with favorable responses, the median progression-free survival (PFS) of 1109 days and overall survival (OS) of 1280 days were significantly better than the median PFS (*p* < 0.00001) and OS (*p* < 0.00001) of the non-responders among the 89 patients or the 77-patient efficacy population. *Conclusion*. Intratumorally administered OT101 exhibits clinically meaningful single-agent activity and induces durable CR/PR/SD in *R/R* HGG patients.

## 1. Introduction

The prognosis of high-grade gliomas (HGG) has not significantly improved despite recent advances in neurosurgery, chemotherapy, immune-oncology, and radiation therapy [1,2,3,4,5,6,7,8,9]. The average overall survival of patients with glioblastoma multiforme (GBM) is merely 10–12 months [10]. Most patients experience the recurrence or progression of their disease within 12 months after frontline therapy and face a dismal outcome with no effective therapy [11,12,13]. Therefore, effective salvage therapies are needed for recurrent/refractory HGG patients who have failed their first line standard therapy.

Transforming growth factor beta 2 (TGFβ) is being explored as a therapeutic target for the treatment of HGGs, including GBM [14,15,16,17,18,19,20,21,22], in part owing to the compelling evidence that the amplified activity of the TGFβ-SMAD signaling pathway contributes to the malignant phenotype and poor prognosis of GBM in adult patients by enhancing tumor growth, invasion, and angiogenesis, as well as compromised immune surveillance [14,15,16,17,18,19,20,21,22]. Notably, the tumor microenvironment (TME) in GBM displays a T-cell exhaustion signature and pronounced T-cell hypo-responsiveness and TGFβ2 has been implicated as a key contributor to the immunosuppressive landscape of the TME in high-grade gliomas [23,24] especially during the later stages of disease progression [15]. OT101 (also known as trabedersen), a TGFβ2-specific synthetic antisense oligodeoxynucleotide (S-ODN), is a first-in-class RNA therapeutic with a Food and Drug Administration (FDA) orphan drug designation (ODD) and pediatric rare disease designation, that is designed to abrogate the tumor-promoting and immunosuppressive actions of TGFβ2 [16,17,25,26]. OT101 has been shown to (i) reduce TGFβ2 production/secretion, (ii) inhibit proliferation and invasive migration, and (iii) enhance sensitivity to the lymphocyte-mediated cytotoxicity of human HGG cells [16,17,25,26]. The intracerebral infusion of OT101 has been shown to result in the rapid distribution of OT101 from the infusion site to other parts of the brain, including the remaining cerebrum, cerebellum, pineal body, and spinal cord in preclinical studies [27]. The feasibility of the intratumoral application of OT101 for treatment of recurrent/refractory HGG patients was first shown in Phase I clinical trials that also provided early activity signals [17,25]. The preliminary findings of the Phase II study NCT00431561 have provided the proof of concept that OT101 can be intratumorally administered using a convection enhanced delivery (CED) platform for up to five months with a favorable safety profile and results in early disease control at six months at a rate comparable to that achieved with temozolomide [28].

Here, we are now reporting our first comprehensive post-hoc analysis of the clinical response data from the Phase II study NCT00431561 in recurrent/refractory supratentorial HGG patients with prolonged follow-up regarding the safety and efficacy of OT101 along with a multivariate analysis of predictive parameters for favorable overall responses and survival outcome. Intratumorally administered OT-101 via CED as second line therapy exhibited clinically meaningful single-agent activity and induced durable complete response (CR), partial response (PR) and stable disease ≥6 months in recurrent and/or refractory (*R/R*) HGG patients. In patients with objective responses, OT101 displayed a previously not reported pattern of single agent activity that was characterized by a delayed onset of robust tumor reductions and, very often, also by an early onset transient increase of tumor edema, which preceded the tumor reductions and objective responses. To our knowledge, this is the first demonstration that the intratumoral delivery of an RNA therapeutic or an immuno-oncology drug candidate as a single agent via extended CED can result in >3 year progression-free survival (PFS) in more than a third of the poor prognosis HGG patients in the efficacy population.

## 2. Materials and Methods

### 2.1. Investigational Medicinal Product and CED Drug Delivery System

The OT101/CED platform and related intellectual property, as well as databases, were acquired from Isarna Therapeutics. The data used in this post-hoc analysis were obtained during the trial registered at ClinicalTrials.gov: NCT00431561. During the NCT00431561 trial, randomized groups of two OT101 dose cohorts received OT101 via a single catheter that was implanted into the target lesion, and OT101 was infused intratumorally using CED (see Supplemental Methods and Figure S1). One treatment cycle with OT101 lasted 14 days and involved 7-days of continuous infusion of OT101, followed by 7-days continuous infusion of isotonic saline solution. OT101 dissolved in isotonic (0.9%) aqueous sodium chloride solution at a final concentration of either 10 or 80 µM was administered at 4 µL/min for 7 days. The total OT101 dose per cycle was 2.5 mg (10 µM group) or 19.8 mg (80 µM group). Patients were to be treated with OT101 for at least 8 weeks corresponding to a minimum of 4 cycles of OT101 and to receive a maximum of 11 treatment cycles of OT101.

### 2.2. Patient Characteristics and Execution of the Clinical Trial

NCT00431561 was a multi-national, multi-center, open-label interventional clinical study on patients with *R/R* HGG (see Supplemental Methods). In order to be eligible for the study, patients had to be consenting adults with a *R/R* HGG (Grade III anaplastic astrocytoma (AA), Grade IV GBM) with supratentorial localization and a measurable target lesion. The diagnosis was histopathologically confirmed before the start of treatments. Patients had to have an expected life expectancy of ≥3 months and a baseline Karnofsky Performance Status (KPS) score ≥70%. Patients who had recent tumor resection within 14 days prior to study entry were excluded, as were patients receiving radiation therapy within 8 weeks prior to randomization. Treatment with chemotherapy, hormone therapy, or any other therapies with established or suggested antitumor effects had to be finished 4–6 weeks (nitrosoureas only) before randomization. No prior stereotactic radiosurgery, interstitial brachytherapy, TGFβ2-targeting therapy, or antitumor vaccination were allowed. Patients who had received another investigational agent within 30 days prior to randomization were not eligible. In order to isolate the clinical single agent anti-HGG activity of OT101, no other cancer treatments, standard or experimental (including but not limited to radiation therapy, chemotherapy, or immunotherapy) were administered unless the patient experienced a progression of disease (PD). Ninety-eight patients (AA: 30; GBM: 68) were randomized to one of the 2 treatment arms of OT101 representing 2 different dose cohorts, namely 2.5 mg/cycle (*N* = 48) and 19.8 mg/cycle (*N* = 50), respectively (Tables S1 and S2). Eight patients discontinued the study after randomization but before the implantation of the catheter-port system. Ninety patients (safety population/SP) who underwent surgery for catheter implantation for OT101 and were randomized to one of 2 dose cohorts of OT101 were evaluable for safety. One patient assigned to the low dose cohort discontinued the study after the surgical procedure but before receiving any OT101 due to procedure related complications. As detailed in Supplemental Methods, there were 89 patients (AA:27; GBM: 62) who had received any amount of OT101 (modified intent-to-treat/mITT population) and, of these, only 77 (efficacy population) (GBM: 51; AA: 26) received at least the intended minimum number of 4 (median: 7, range: 4–11, mean ± SE: 9.8 ± 0.3) OT101 treatment cycles (Tables S1 and S2). The mITT population included 25 females and 64 males at a median age of 45 (range: 19–73; mean ± SE = 46.3 ± 1.3) years with a median baseline KPS score of 90 (range 70–100; Mean ± SE: 87.6 ± 0.9). Patient characteristics and the neuro-oncologic medical history of the patients are shown in Table 1. Fifty-eight patients were Caucasian, whereas 31 were Asian. 62 patients had GBM, and 27 had AA. Forty patients were treated at the low dose level (10 µM concentration in the infusate; 2.5 mg/cycle), and 49 patients were treated at the high dose level (80 µM concentration in the infusate; 19.8 mg/cycle) of OT101. The mean size of the target lesion for the mITT population was 9.3 ± 0.6 cm^2^ for 2-D surface area measurements and 27.1 ± 2.5 cm^3^ for 3-D volume measurements. Sixty-eight patients (78.2%) had a single measurable contrast-enhancing lesion and non-measurable contrast-enhancing lesions were reported only in 20 (22.5%) patients (Table 1). The median time from first diagnosis to randomization was 229 (mean ± SE: 379 ± 59) days and the median time from last cancer therapy to randomization was 103 (mean ± SE: 248 ± 53) days. Patients received 7.0 ± 0.3 (range: 1–11; median: 6) cycles of OT101 at a total cumulative dose of 45.2 ± 4.6 (median: 22.7, range: 1.1–152.1) mg/m^2^.

### 2.3. Ethics Statement and Study Approval

This was a post-hoc analysis of the primary study data from NCT00431561 Phase IIb clinical study [28]. No human subjects were involved in this analysis.

### 2.4. Safety and Efficacy Measurements

As detailed in Supplemental Methods, safety analyses were performed for all 90 patients in the safety population (SP). Efficacy analyses were performed for all 89 patients in the mITT population and the 77-patient primary efficacy population. For immediate decision-making during the course of the study, the local neuroradiologists evaluated patients’ local MRIs according to study-specific procedures as filed in the trial master file (TMF). For a standardized response assessment, an independent central MRI reading (CMRIR) was performed by a specialized central reading institute (Timaq Medical Imaging Inc, Zurich, Switzerland). The central reading was conducted by two independent neuroradiologists as readers with an additional adjudicator for cases of predefined discrepancies in the reports of the two readers (see Supplemental Methods). Best overall response (BOR) was defined as the best response documented from randomization until the progression of disease (PD). For determining the treatment response of individual patients to OT101, standard Macdonald criteria were used (see Supplemental Methods). MRI scans were performed according to protocol requirements, initially every 4 weeks (namely, at baseline, prior to cycle #’s 3, 5, 7, 9, 11, final visit in week #25) until the end of treatment visit in Week 25, then every 4 weeks X 2, every 8 weeks X 8, every 3 months X 2, and subsequently every 6 months. Tumor size determinations were made both in 2-D (two-dimensional: product of the two longest perpendicular diameters) and in 3-D (three dimensional: product of the longest perpendicular diameters in one plane and the longest orthogonal diameter to that plane). In this analysis, we retrospectively identified patients with pseudoprogression (PsP) using a modification of the criteria reported by Taal et al. [1]. Specifically, such patients (1) must have experienced an MRI-documented >25% increase in the size of their contrast enhancing target lesion within 2 months after the initiation of treatment in the absence of clinical deterioration, the worsening of neurologic status or an increased need for steroid use and (2) must have subsequently experienced an MRI-documented >50% decrease in the size of their contrast-enhancing target lesion along with stable or improved clinical status for more than 6 months in the absence of new anti-cancer therapy or increased steroid use for management. Overall survival (OS) was the time from the date of randomization to time of death. Surviving patients were censored at their last follow-up. Progression-free survival (PFS) was the time from randomization to documentation of PD or death. Patients who remained alive without PD were censored at last follow-up. Standard definitions were used for time to progression and duration of objective response (see Supplemental Methods).

### 2.5. Statistical Analyses

Standard statistical methods were applied for the analysis of data (see Supplemental Methods). The distribution of time-to-event survival end points on the OS and PFS curves were estimated by the Kaplan–Meier method. Differences between patient subgroups were evaluated by log-rank statistics. The analyses were performed using JMP software (version 10.02, SAS Institute, Inc, Cary, NC, USA), and R software, version 3.5.2 (R Foundation for Statistical Computing, Vienna, Austria), loaded with statistical packages for survival analysis (survMisc_0.5.5; survival_2.44–1.1 and survminer_0.4.4) with default settings). A generalized linear model (GLM; binomial distribution; logit function link function) was fitted to explore several clinical parameters as potential predictors for best overall response (BOR). The parametric survival model was fitted the time to death using linear regression to predict improved OS. The best fit model was chosen utilizing both the Akaike information criterion and the Bayesian information criterion from assessing Weibull, lognormal, exponential, Fréchet, and loglogistic distributions of the survival probabilities (JMP 10.02, SAS, Cary, NC, USA).

## 3. Results

### 3.1. Safety of the Drug Delivery System and Intratumoral OT101 Treatments in R/R HGG Patients

Ninety patients (safety population/SP) who underwent surgery for catheter implantation for OT101 and randomized to one of two dose cohorts of OT101 were evaluable for safety (Table S1; see Methods and Supplementary Materials). Complications related to CED catheter placement and/or use were encountered, including one fatal outcome due to intracerebral hemorrhage prior to initiation of OT101 treatments (Tables S2–S4). Eleven patients (12.2%; three cases of brain abscess, three cases of meningitis (one fatal case with concomitant sepsis), four cases of surgical wound inflammation/infection, and one case of the inadvertent administration of gadolinium via the CED catheter) experienced surgical procedure/device-related or possibly related serious adverse events (SAE) associated with the implantation of the intratumoral catheter and use of the CED system that led to the permanent discontinuation of OT101 protocol therapy (see Supplementary Materials, Table S4). Intratumorally administered OT101 exhibited a promising safety profile, as documented in Table S2 and Tables S5–S10; see Supplementary Materials). Most of the adverse events (AE) or SAE were not related to OT101 but rather to neurological disorders (e.g., increased intracranial pressure or brain edema) associated with the underlying HGG and/or its progression. Among the 90 patients in the safety population, 10 (11.1%) experienced OT101-related/possibly-related Grade 3 or 4 AE (Table S5), and only two patients (2.2%) experienced OT101-related SAE that led to the discontinuation of therapy (Table S6, Table S10).

### 3.2. Single Agent Anti-HGG Activity of Intratumorally Delivered OT101 as Measured by Best Overall Response

As detailed in the Methods and Supplemental Methods, efficacy analyses were performed for the 89 HGG patients (AA: 27; GBM: 62; mITT population) who received any amount of OT101 and the efficacy population (EP) of 77 HGG patients (GBM: 51; AA: 26) who received at least the intended minimum number of four (median: 7, range: 4–11, mean ± SE: 9.8 ± 0.3) OT101 treatment cycles (Tables S1, S8 and S9). Patient characteristics and neuro-oncological medical history of the mITT population are shown in Table 1. None of the 12 patients who received only 1–3 cycles of OT101 had an objective response or a meaningful stabilization of disease progression. Other than the number of OT101 cycles and cumulative OT101 dose received, there were no significant differences between the mITT and efficacy populations (Table S8). By comparison, of the 77 patients in the efficacy population receiving 4–11 cycles of OT101, 19 (GBM: 5, AA: 14) achieved durable objective responses (CR: 3, PR: 16) and seven (GBM: 5, AA: 2) had a stable disease (SD) lasting ≥6 months (Table S9).

**Figure 1:**
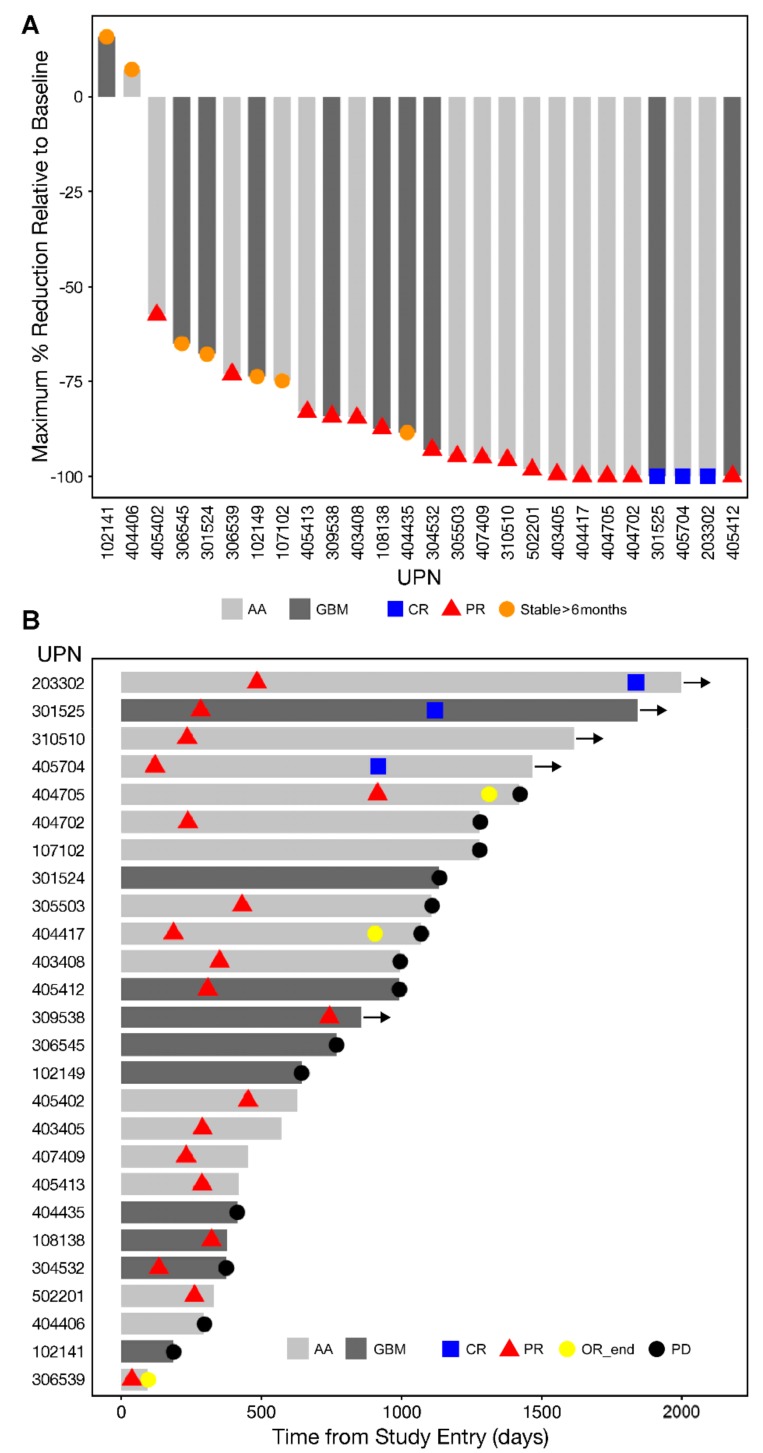
(**A**) Waterfall plot of Maximum Tumor Reductions of the 26-patient Favorable Response Population: A waterfall plot is depicted to show the maximum tumor reduction relative to baseline (% reduction of the 3-D volume of the target lesion) in each of the 19 recurrent and/or refractory (*R/R*) high grade glioma patients who had a complete response (CR), partial response (PR) or stable disease lasting ≥6 months to OT101 single agent therapy. Vertical bars on these plots measured maximum reduction in tumor volumes of the treated target lesions following OT101 treatment. The best overall response (BOR) for each patient are indicated with specific symbols. (**B**) Swimmer Plot of Best Overall Responses of the 26-patient Favorable Response Population: The onset and duration of CR/PR, end of the objective response (OR) and onset of progression of disease (PD) are indicated with specific symbols. In patient 404705, the PD was an unconfirmed single point determination based on a follow-up MRI. In patient 3100510, the BOR determination was based on local review of the MRI data; the BOR was based on central review of MRI scans in others. See Table S9 for additional details.

**Figure 2:**
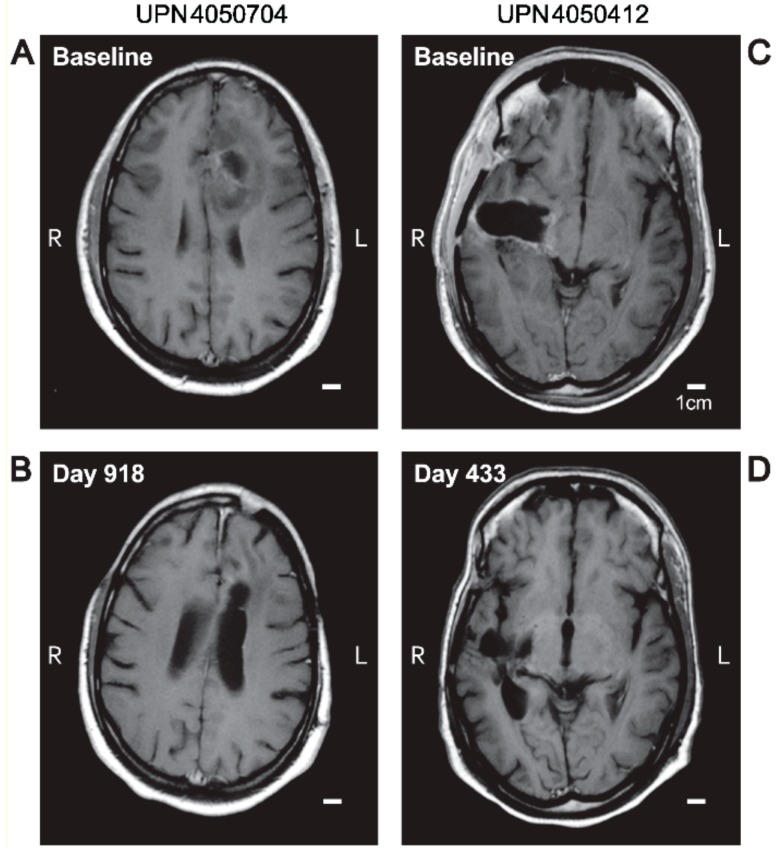
MRI-response of target lesion in OT101 treated *R/R* high-grade glioma patients. Depicted are T1-weighted spin echo (SE) post-contrast axial MRI images at baseline vs. post-treatment with OT101 at 433 days post randomization to their respective OT101 dose cohorts. Panels **A** and **B**: Unique patient number (UPN) 405-0704 (anaplastic astrocytoma (AA), WHO Grade III) achieved a CR. Panels **C** and **D**: UPN4050412 (glioblastoma multiforme (GBM), WHO Grade IV) achieved a PR.

The objective responses were preceded by a slow but robust size reduction of the target lesions in both GBM and AA patents (Figure 1 and Figure 2 and Figures S2 and S3). Figure 2 and Figure S4 depict representative MRI images from two GBM patients and one AA patient who had an objective response. Waterfall plots depicting the maximum % and log_10_ reduction values for the tumor volumes of the objective responders following OT101 treatment are shown in Figure 1A and Figure S5, respectively. The maximum percent reduction (mean ± SE) of the 3-D volume of the treated target lesion for these 19 objective responders was 91.9 ± 2.6 % (median = 95.7%, range: 57.5–100%) (Figure 1A). The average (mean ± SE) time for 99% (2-logs) reduction in tumor volumes for the 19 responders was 31.19 ± 6.47 (median = 23.3, range: 9.9–115.4) months, and the average for 90% (1-log) reduction was 15.6 ± 3.2 (median = 11.7: range: 4.9–57.7) months (Figure S5). Six patients achieved 100% reduction in 3-D tumor volume of the target lesion over the course of the treatment, as shown in the waterfall plot depicted in Figure 1A. Like the 19 patients who had a CR or PR as their best overall response, five of the seven patients with an SD ≥ 6 months also had marked tumor reductions after treatment with OT101 (Figure 1A).

The onset and duration of the CR or PR in the 19 patients with objective responses as well as the duration of the SD in the seven patients who had an SD ≥6 months as their BOR is illustrated by the swimmer plot depicted in Figure 1B. Specifically, 19 patients (“objective responders”) achieved a PR with a time of onset at a median of 287 days (Range: 37–914 days) (Figure 1B). The PR in three of these patients deepened to a CR at 917, 1120 and 1838 days, respectively (Figure 1B and Tables S9 and S10). Of these 19 objective responders, 10 had a response duration of >6 months, including four patients with a response duration of 6.1–9.4 months (UPN 532, 409, 417, and 405) and six patients had a durable response with a median response duration of 3.8 years (viz.: 1.1 years [UPN705], 3.0 years [UPN702], 3.7 years [UPN704], 3.8 years [UPN510], 4.2 years [UPN402], and 4.3 years [UPN525]) (Figure 1B and Table S9). Seven of these 19 patients developed a PD at a median of 1070 (range: 374–1423) days (mean ± SE = 1035 ± 109 days).

We next sought to identify predictive variables associated with favorable response to OT101. All of the 26 patients (100%) who favorably responded to OT101 (viz.: 19 patients with CR/PR and seven patients with an SD ≥ six months) received ≥ 4 treatment cycles compared to 51 out of 63 (81%) non-responder patients (26/26 vs. 51/63; Fisher’s exact test, two-tailed, *p*-Value = 0.016). A greater proportion of the favorable responders had AA than the entire mITT population (16/26 (AA for responders) vs. 27/89 (11/63 AA for non-responders), Fisher’s exact test, two-tailed, odds ratio = 7.35, *p*-Value = 8.7 x 10^−5^). The group with favorable responses showed a strong trend for having been treated with higher cumulative total doses of OT101 than the cumulative doses received by the non-responders (*p*-Value = 0.06). The 26 patients favorable response group had a significantly higher proportion of patients with baseline KPS scores of 90–100 than the 63 patients in the non-responder group (24/26 vs. 40/63; Fisher’s exact test, two-tailed, odds ratio = 6.8, *p*-Value = 0.008). Forty-three patients (19 [47.5%] in the 2.5 mg dose cohort and 24 [49%] in the 19.8 mg dose cohort) received systemic corticosteroids after randomization for the treatment of brain edema. A comparison of corticosteroid treatment patterns revealed highly significant differences between the 26-patient group with favorable responses vs. the non-responders. Eighteen of 25 (72%) evaluable patients in the favorable response group but only nine of 59 (15%) of evaluable non-responders within the mITT population had received either no dexamethasone or very limited amounts of dexamethasone for treatment of specific AE only (G-Test, G = 33.7, df = 4, *p* < 0.0001). A greater (albeit only at a statistical trend level) portion of the evaluable 24 patients within the 26-patient group with favorable responses had failed after first-line therapy radiation alone or in combination with the temozolomide in first-line vs. other radiation plus other chemotherapy agents (paclitaxel, nitrosoureas, carboplatin, topoisomerase inhibitors, and anthracyclines) than 54 evaluable patients within the 63-patient non-responder population (13/24 [54%] vs. 19/54 [35%], G = 6.99. *p* = 0.07). In a generalized linear model (GLM)-based univariate analysis, age (Z-statistic: 2.7, *p* = 0.006), KPS score (Z statistic: 2.5, *p* = 0.01, diagnosis (AA vs. GBM) (Z-statistic: 3.9, *p* = 0.0001) and dexamethasone use (Z-statistic: 4.8, *p* < 0.00001) emerged as significant predictors of a favorable response.

We next used a GLM-based multivariate analysis to further evaluate the predictive value of clinical parameters for favorable clinical responses to OT101 therapy. The final GLM (binomial distribution, χ^2^ = 39.7, df = 12, *p* < 0.0001) for the 77-patient efficacy population contained seven predictor variables that were available from 67 of the 77 patients: baseline KPS score, composition of previous therapy, cancer subtype, dexamethasone use, total OT101 dose, age, and baseline 3-D tumor volume for the target lesion. Among these seven variables, three significant predictors were identified: baseline KPS score (χ^2^ = 6.4, *p* = 0.011), histopathologic diagnosis (AA vs. GBM) (χ^2^ = 5.7, *p* = 0.017) and dexamethasone use (χ^2^ = 14.2, *p* = 0.0067) (Table S11). In this model, total OT101 dose administered approached statistical significance (χ^2^ = 3.7, *p* = 0.055). These analyses were also performed for 77 evaluable patients within the 89-patient mITT population (binomial distribution, Chi-square value = 43, df = 12, *p* < 0.0001), and the results were consistent with those obtained for the efficacy population: Three significant predictor variables were identified: baseline KPS score (χ^2^ = 5.8, *p* = 0.016), cancer subtype (χ^2^ = 6.4, *p* = 0.012), and steroid/dexamethasone use (χ^2^ = 16.6, *p* = 0.002). Hence, being an AA rather than a GBM patient, having a higher baseline KPS score, receiving either no dexamethasone or short-term dexamethasone during the management of AE are associated with more favorable responses for *R/R* HGG patients both within both the efficacy and mITT populations treated with OT-101 as a single agent.

### 3.3. Edema and Pseudo-Progression in OT101-Yreated Target HGG Lesions

**Figure 3:**
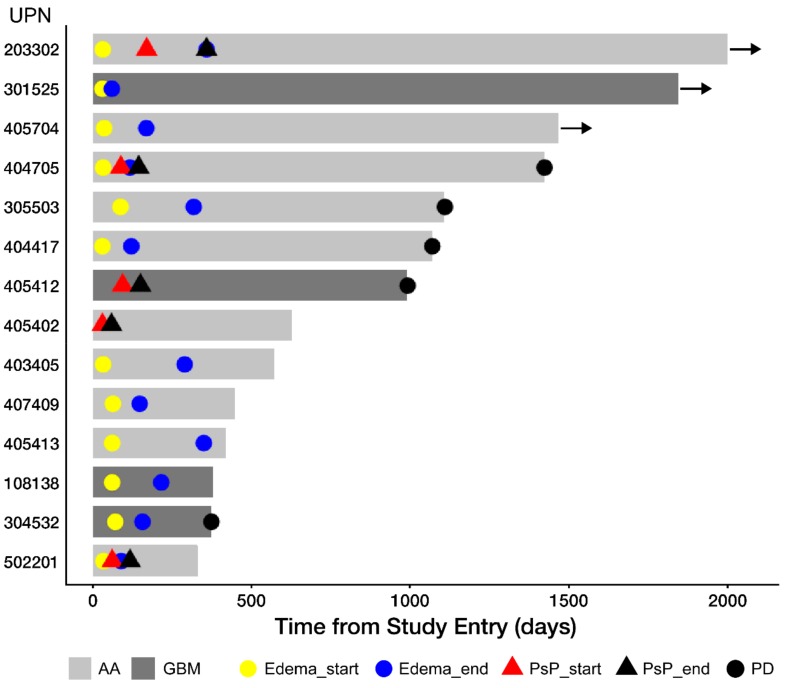
Swimmer plot for onset and duration of intratumoral edema and/or pseudoprogression for 14 patients with CR or PR as their BOR. The onset and end of intratumor edema, pseudo-progression and onset of PD are indicated with specific symbols. In patient 404705, the PD was an unconfirmed single point determination based on day 1422 follow-up MRI.

**Figure 4:**
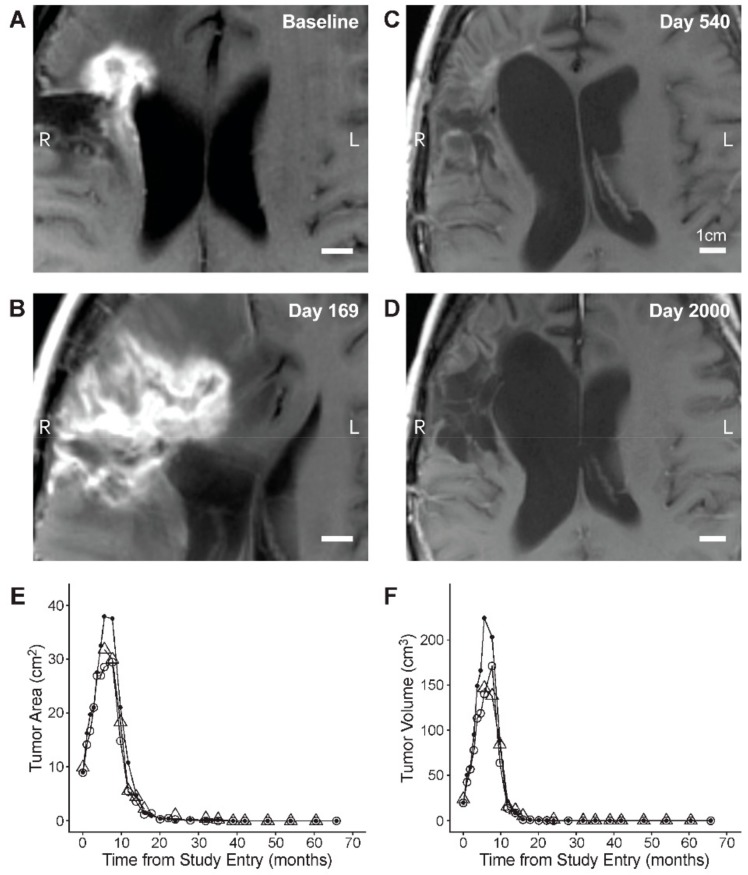
Pseudo-progression and CR in *R/R* AA (WHO Grade 3) patient, UPN203302. (**A**–**D**): Depicted are T1-weighted spin echo (SE) axial MRI images obtained at baseline and at the indicated time points after randomization to the 2.5 mg/cycle dose cohort of OT101. See Figure S6 for details provided for the same images in smaller magnification. (**E** and **F**): Review of MRI images by two-to-three independent reviewers (open circle: Reviewer 1; closed circle: Reviewer 2; triangle: Reviewer 3/adjudicator) showed a time-dependent decrease of the 2-D (Panel **E**) and 3-D (Panels **F**) size of the target lesion following a significant increase during the early period of pseudo-progression.

By using the independent central reviews of the MRI data, we next sought to determine if there were any clinical surrogate markers of an inflammatory response in OT101-treated tumors. In 12 of the 19 (63.2%) objective responders, OT101 caused within 2–3 months after initiation of intratumoral infusions new or increased edema in the target lesions which was transient (Time to onset: 34 [range: 30–87] days; time to end: 162 [range: 59–358] days (Figure 3). Notably, five patients, including three of the afore-mentioned 12 with OT101-induced tumor edema, experienced pseudo-progression (PsP) (time to start: 88 [range: 30–169] days; and time to end: 144 [range: 59–358] days (Figure 3 and Figure 4, and Figure S6). No clinical deterioration was observed, and no corticosteroids were used for management of PsP in any of these patients whose median PFS and OS were 899 days (range: 331–2000 days) and 1467 days (range: 1069–2000 days), respectively. Hence, 14 of the 19 (73.7%) patients who had a CR or PR as their BOR had MRI changes consistent with OT101-associated inflammatory responses involving the treated target lesions.

### 3.4. PFS and OS Outcomes After OT101 Therapy

**Figure 5:**
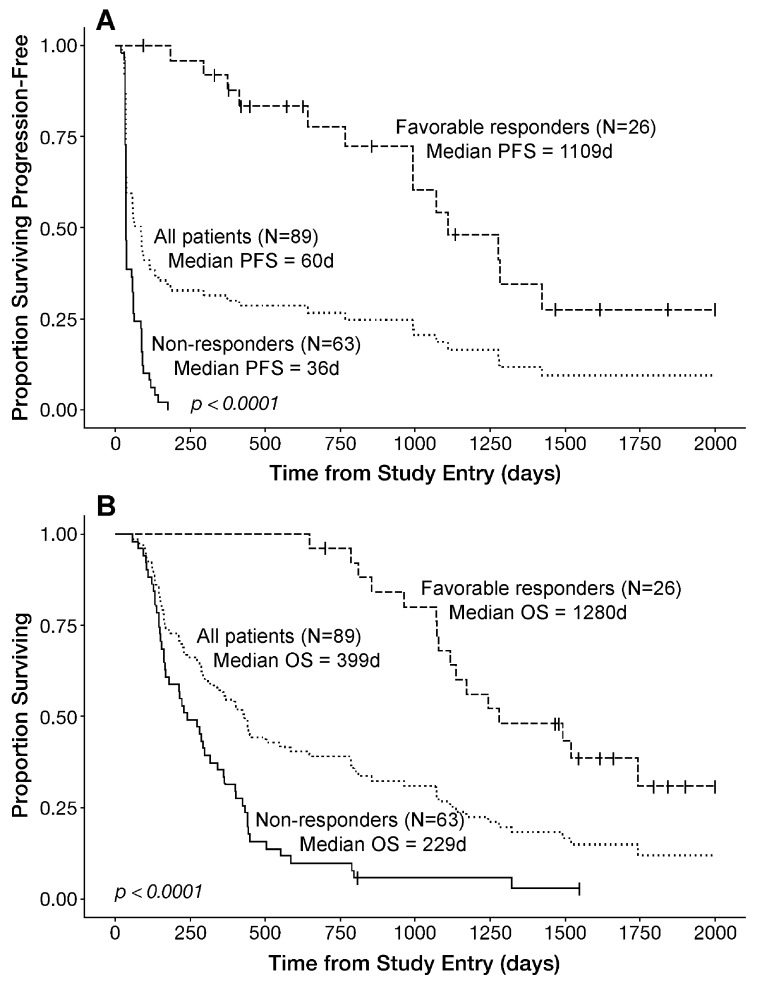
Survival Outcome of HGG Patients According to Their Best Overall Responses to OT101. (**A**): progression-free survival (PFS) outcome of the mITT population. Favorable BOR of CR, PR or stable disease (SD) ≥ 6 months is associated with an improved PFS in *R/R* HGG patients treated with OT101 monotherapy. Depicted are the PFS curves of the entire 89-patient mITT population as well as 26 favorable responders and 63 non-responders. Patients received no other cancer therapies during the depicted PFS. See also Figure 1 and Table 2. (**B**) OS outcome of the mITT population. Favorable BOR of CR, PR or SD ≥ 6 months is associated with an improved OS in *R/R* HGG patients treated with OT101 monotherapy. Depicted are the OS curves of the entire 89-patient mITT population as well as 26 favorable responders and 63 non-responders. See also Table 2.

The Kaplan–Meier probability estimates for the median PFS and OS of the mITT population of 89 patients of OT101 therapy were 60 days (95% CI: 39–93) and 399 days (95% CI = 283–503), respectively (Table 2). The median PFS for the efficacy population was 86 (95% CI: 40–134) days, which was significantly better than the median PFS of 32 (95% CI: (32–NA) days for the 12 patients treated with <4 cycles of OT101 (log-rank χ^2^ = 16.1, log-rank *p*-Value < 0.0001) (Table 2). Likewise, the median OS of the efficacy population was significantly better than the median OS for the remaining 12 patients in the mITT population receiving less than four cycles of OT101 (432 [95% CI: 299–788] days vs. 128 [95% CI: 93–NA] days, log-rank χ^2^ = 19.5, log-rank *p*-Value < 0.0001) (Table 2). Notably, the median PFS and OS of the 26 patients with favorable responses were significantly better than the PFS and OS of the 63 non-responders (median PFS: 1109 days vs. 36 days, *p* < 0.00001; median OS: 1280 days vs. 229 days, *p* < 0.00001) (Table 2 and Figure 5). Their PFS and OS outcomes were also significantly better than the PFS and OS outcomes of the remaining 51 patients within the efficacy population (Table 2 and Figure S7).

### 3.5. Univariate and Multivariate Analysis of Potential Predictors of PFS and OS Outcome After OT101 Monotherapy

As potential predictors of improved PFS and/or OS, we evaluated several categorical variables using Kaplan–Meier estimates. In addition, the significance of continuous predictor variables for OS was evaluated utilizing a two arm Kaplan–Meier analysis, whereby the PFS/OS outcome of the top third of the patients for a given variable was compared to the PFS/OS outcome of the bottom third of the patients for that variable. In our initial univariate analysis, the histopathologic diagnosis of AA (vs. GBM), younger age, better performance status (as measured by KPS score), higher number of OT101 cycles received, no or very limited dexamethasone use, as well as favorable response (as defined by BOR of CR, PR or SD ≥6 months) have emerged as strong predictors of better PFS and OS outcomes after OT101 therapy (Table 3 and Figure S8). In the univariate analysis, the observed differences in PFS (log-rank χ^2^ = 72.6, *p* < 0.00001) and OS (log-rank χ^2^ = 51.6, *p* < 0.00001) outcomes between the 26 patients with favorable responses vs. 63 non-responders were highly statistically significant (Table 3 and Figure 5). Furthermore, higher cumulative OT101 doses appeared to correlate with better PFS and OS outcomes (Table 3). The median OS for the 30 patients representing the top third of at risk patients within the mITT population relative to the total dose of OT101 (range: 53–152 mg/m^2^) was 447 (95% CI: 402–1079) days and showed a trend towards better outcome (log-rank χ^2^ = 3.5, *p*-Value = 0.06) when compared to the median OS of 221.5 (95% CI: 152–406) days for the 30 patients representing the bottom third of at risk patients (range: 1.1–14 mg/m^2^) (Table 3).

Multivariate analyses performed in a parametric model established favorable response as the overwhelming predictor of the PFS and OS outcome after adjustment for the effects of other covariates (*p* < 0.00001) (Table 3 and Table S12). Dexamethasone use, total dose of OT101 and histopathological diagnosis were significant additional predictive variables for the PFS and OS outcome after adjustment of the effects of the favorable response (Table 3 and Table S12). Multivariate analyses were also performed in a second parametric model that did not include the favorable response as a covariate. Histopathological diagnosis (Z-statistic = 3.9, *p* = 0.0001), KPS score (Z-statistic = 2.6, *p* = 0.009) and dexamethasone use (Z-statistic = 5.4, *p* < 0.00001) were significantly related to PFS outcome. Likewise, histopathological diagnosis (Z-statistic = 3.9, *p* = 0.0001), KPS score (Z-statistic = 3.3, *p* = 0.0009) and dexamethasone use (Z-statistic = 5.1, *p* < 0.00001) were also significantly related to OS outcome (Table S12).

To build a multivariate predictive model for the OS outcome after OT101 therapy that could be used to inform the design of future clinical trials and does not include favorable response as a covariate, we also investigated the effect of each of the clinical parameters controlling for cancer subtype (GBM or AA) by utilizing a two-factor parametric model. Age and cancer subtype in the two-factor model were both independently significant predictors of OS (subtype: *p* < 0.0001; age: *p* = 0.009), whereby GBM and increasing age resulted in reduced survival probabilities, and their effects were maintained regardless of the other parameter. Steroid use was also a significant factor (categorical variable, *p* = 0.0004) regardless of cancer subtype as was the number of treatment of cycles (continuous variable, *p* < 0.0001; whereby increasing the number of cycles was associated with improved OS. The dose level of OT101 and previous therapy were not significant predictors, whereas the total cumulative dose of OT101 maintained a strong trend towards a better OS outcome with increasing total dose (continuous variable, *p* = 0.07). Baseline tumor size also exhibited a moderate trend of increasing tumor size being associated with worse OS (continuous variable, *p* = 0.11) regardless of cancer subtype. Gender, ethnicity, or the hematological parameters at baseline when evaluated in conjunction with cancer subtype in these two-factor models were not significant predictors for the OS outcome. The two-factor model was expanded to include additional clinical parameters to fully characterize independent predictors of OS for OT-101 treated patients. The final multivariate parametric OS model (loglogistic best fit highly significant with a Chi-square value = 57.2, df = 12, *p* < 0.0001) model contained seven predictor variables measured from 77 evaluable patients within the 89-patient mITT population: baseline KPS score (70–80 versus 90–100), composition of previous therapy, cancer subtype, steroid use, total OT-101 dose, age, and baseline 3-D tumor volume for the target lesion. Three significant predictors were identified: baseline KPS score (χ^2^ = 10.1, *p* = 0.0015), cancer subtype (χ^2^ = 6.06, *p* = 0.014) and steroid use (χ^2^ = 18.9, *p* = 0.0008). Being an AA rather than a GBM patient, having a higher baseline KPS score, and receiving either no steroids or short-term steroids during the management of AE were associated with improved OS for *R/R* HGG patients treated with OT-101 as a single agent. The same conclusions were reached when the analyses were limited to the 67 evaluable patients in the 77-patient efficacy population: Chi-square value = 56, df = 12, *p* < 0.0001; three-predictors: baseline KPS score (χ^2^ = 12.6, *p* = 0.0004), cancer subtype (χ^2^ = 4.4, *p* = 0.036) and steroid use (χ^2^ = 16.7, *p* = 0.002).

## 4. Discussion

Our post-hoc analysis of the NCT00431561 Phase II clinical study outcome data demonstrate that the anti-TGFβ2 RNA therapeutic OT101 exhibits clinically meaningful single-agent activity in the absence of other anti-cancer drugs or radiation therapy and induces durable CR, PR and SD in more than one third of the efficacy population (viz.: 26 of 77 [33.8%]) comprised of *R/R* HGG patients when intratumorally administered via CED. Notably, favorable responses were observed in both GBM and AA patients (GBM: 10 of 26 [38.5%]; AA: 16 of 26 [61.5%]). In the 19 *R/R* HGG patients who achieved a PR or CR as their BOR, OT101 displayed a previously not reported pattern of single agent activity, which was characterized by (i) slow but robust tumor size reductions (median time for 90% reduction of the baseline tumor volume = 11.7 months); (ii) very often, an early onset transient increase of tumor edema commencing at a median of 34 days and/or pseudo-progression commencing at a median of 88 days after the initiation of therapy which preceded the tumor size reductions; and (iii) late-onset objective responses that were achieved at a median of 287 days. Notably, the >3 year median PFS and >3.5 year OS of the 26 patients with favorable responses were significantly better than the PFS and OS of the 63 non-responders (median PFS: 1109 days vs. 36 days, *p* < 0.00001; median OS: 1280 days vs. 229 days, *p* < 0.00001). Their PFS and OS outcomes were also significantly better than the PFS and OS outcomes of the remaining 51 patients within the 77-patient efficacy population. To our knowledge, this is the first demonstration that the intratumoral delivery of an RNA therapeutic (or any other immuno-oncology drug candidate) via extended CED in the absence of other therapeutic agents or radiation results in >3 year median PFS and >3.5 year OS in greater than one third of the efficacy population in a *R/R* HGG trial.

TGFβ has been shown curb the anti-tumor function of TME by both limiting T-cell infiltration and suppressing the function of the immune system elements [29,30,31,32]. TGFβ has recently been implicated in T-cell exclusion from TME contributing to tumor immune evasion and a poor response to immune checkpoint inhibitors [29,30,31,32]. TGFβ inhibition combined with immune checkpoint blockade has been shown to induce responses of otherwise PD-1/PD-L1 unresponsive tumors [29,30,31,32]. Tauriello et al. recently demonstrated that inhibition of TGFβ signaling with galunisertib can contribute to sustained anti-tumor immunity and PD-1/PD-L1 responsiveness in preclinical animal models [29]. These recent observations regarding the role of TGFβ in tumor-induced immune suppression prompt the hypothesis that OT101 could provide the basis for a combined treatment strategy of targeting both TGFβ and PD-1/PD-L1 pathways in recurrent HGG patients. In the present study, no or minimal dexamethasone use was associated with better survival outcomes. These findings suggest that lifting the immune suppression with TGFβ-targeting agents like OT101 may be most beneficial to those HGG patients with a less compromised immune system and underscore the importance of restricting the use of dexamethasone to only those cases with symptomatic brain edema.

No integrated laboratory studies were performed during the primary study to further elucidate the immunomodulatory effects of OT101 and its molecular mechanism of action. We hypothesize that the observed intratumoral edema as well as PsP among the favorable responders to OT101 may be the result of the inflammatory response of glioma cell death induced by OT101-induced TGFβ2 downregulation perhaps exacerbated by OT101-induced abrogation of the immunosuppressive actions of TGFβ2 in the TME. It is noteworthy that Han et al. recently reported that the downregulation of TGFβ2 with adenoviruses delivering short hairpin RNAs (shRNAs) leads to tumor cell death by inducing apoptosis signal-regulating kinase 1 (ASK1) activation and p38 and c-Jun N-terminal kinase (JNK) phosphorylation through an endoplasmic reticulum (ER) stress [33]. ASK1 has been identified as the key mediator in TGFβ2 downregulation-induced death that induces cytotoxic tumor cell death via p38/JNK activation and (or) induction of ER stress [33]. In future studies of OT101, we will seek to determine if OT101 treatments result in ASK1 activation and ER stress, and we will evaluate pre- and post-treatment biopsy specimens for evidence of increased lymphocyte infiltration. If confirmed in larger scale clinal trials, intratumoral edema and/or pseudoprogression could also emerge as clinical biomarkers of early response in the context of OT101 treatments or other RNA-targeted therapy.

A number of limitations should be pointed out for this study: First and foremost, our results should be interpreted with due caution within the inherent limitations of a post-hoc analysis. Having a favorable response (CR, PR, or prolonged SD) are not proven surrogate markers for PFS or OS in HGG patients [34]. Furthermore, pseudo-progression (PsP) [35] was also encountered as an imaging confounder for the interpretation of MRI results within the first three months after the initiation of therapy. Furthermore, the presence of an intratumoral catheter created a challenge for the radiographic response determinations until the catheter was removed. Another limitation of this Phase II study was the heterogeneous nature of the patient population. Unfortunately, genomic biomarkers (e.g., isocitrate dehydrogenase gene (IDH)-1/IDH-2 mutations, methylguanine-DNA-methyl transferase (MGMT) gene promoter methylation, and complete deletion of both the short arm of chromosome 1 (1p) and the long arm of chromosome 19 (19q) (1p/19q co-deletion) were not collected during the primary Phase II study. Therefore, this post-hoc analysis does not inform us about how active OT101 might be against IDH-wildtype and/or MGMT-unmethylated HGG patients who have failed standard 1st line therapy with Radiation + TMZ. It will be important to evaluate the clinical potential of OT101 in a more homogenous patient population (e.g., IDH-wildtype patients who have failed a temozolomide-based first line adjuvant therapy, especially those whose tumors are MGMT-unmethylated).

*R/R* HGG, especially *R/R* GBM, has a dismal prognosis with no effective standard therapy. Very limited treatment options available for the salvage treatment of recurrent disease in patients with GBM include treatment with bevacizumab, second-line chemotherapy (e.g., temozolomide, lomustine, procarbazine + lomustine + vincristine (PCV), surgical resection and reirradiation. Notwithstanding the fact that MRI imaging provides only a surrogate measure of the disease activity and status in HGG patients [33], the observed association of the favorable responses with prolonged PFS and OS demonstrates that OT101 treatments can benefit *R/R* HGG patients, and the BOR is a predictor of OS outcome for *R/R* HGG patients treated with intratumoral infusions of OT101. Treatment outcome simulations using multivariate predictive modelling of PFS times for AA as well as GBM patients yielded significantly higher median PFS times for favorable responders vs. non-responders (*p* < 0.00001; Figure S9). While standard Macdonald criteria [36] were used in the present study and Response Assessment in Neuro-Oncology (RANO) criteria might have resulted in different PFS outcome results [37], the observed robust tumor reduction in a subgroup of patients with favorable responses as confirmed by independent central review combined with the long-term survival of favorable responders is unprecedented for an immune-oncology drug. The intratumoral infusion of a recombinant polio-rhinovirus chimera has also shown a promising clinical activity signal in a recent clinical study [12]. The combined use of these two distinct modalities may have high clinical impact potential and should be explored.

Safety analyses were performed for all 90 patients in the safety population (SP), which included all randomized patients who underwent the catheter implantation surgery or OT101 treatments. Intratumorally administered OT101 exhibited a promising safety profile. Only 10 (11.1%) experienced OT101-related/possibly related Grade 3 or 4 AE, and only two patients (2.2%) experienced OT101-related SAE that led to discontinuation of OT101. Most of the AE or SAE were not related to OT101 but rather to neurological disorders (e.g., increased intracranial pressure or brain edema) associated with the underlying HGG. In most cases, the investigators regarded progression of the primary disease as the cause for the AEs and SAEs. In the present study, OT101 was administered via CED [38], and several CED procedure and device-related complications were identified. Twenty-eight patients (31.1%) experienced surgical procedure-related (possibly, likely, or definitely related) SAE associated with the implantation of the intratumoral catheter and use of the CED system. The favorable impact of the treatment duration (viz., cycle number) and cumulative total OT101 dose on OS outcome within the mITT population suggests a dose effect—within the confines of a study that was not randomized based on the cumulative total dose or treatment duration—and emphasizes the importance of reducing the risk of procedure/device related early complications, including central nervous system (CNS) infections to not only maximize patient safety but also to further improve the OS outcome. The use of antibiotic-impregnated catheters that have recently become commercially available and the implementation of an intensive training program for the medical personnel involved in the maintenance of the CED system to ensure high standards of hygiene as well as perioperative use of prophylactic antibiotics during catheter and port placement may reduce the risk of infections. Additional risk mitigation measures may help avoid potential catheter misplacement and intratumoral bleeding in future studies by (i) avoiding biopsies from the catheter tip which might lead to tissue disruption as well as bleeding and by (iii) applying high precision catheter placement by an optimized stereotactic procedure and by tracking the delivery of the OT101 infusion using real-time intraoperative MRI imaging as well as using a software for 3-D drug distribution simulation. We anticipate that the incorporation of the insights and lessons learned from this post-hoc analysis in the further clinical evaluation and development of OT101 will help improve the potential for patient benefit. While a CED system has been used to deliver OT101 in clinical trials to date, it may also be possible in the future to administer OT101 without a neurosurgical intervention as a payload in a nanoformulation that can cross the blood-brain-barrier (BBB) via active transport mechanisms employing receptor-mediated or adsorption-mediated endocytosis, including but not limited to poly(lactic-*co*-glycolic acid) (PLGA)-based polymeric nanoparticles and targeted nanoliposomes [39,40]. It may also be possible to achieve therapeutic CNS delivery of OT101 by using clinically available methods of transient and repeated BBB disruption, such as the MRI-guided focused ultrasound-mediated drug delivery platform [41] or low intensity pulsed ultrasound (LIPU) using a cranial ultrasound device that can be activated prior to drug administration at [42,43].

## 5. Conclusions

Our post-hoc analysis of the NCT00431561 Phase II clinical study outcome data demonstrates that the anti-TGFβ2 RNA therapeutic OT101 when intratumorally-administered via CED exhibits promising single-agent activity against *R/R* HGG. Most importantly, OT101 induced durable CR, PR and SD in more than one third of the efficacy population. In the objective responders, OT101 displayed a previously not reported pattern of single agent activity, which was characterized by (i) slow but robust tumor size reductions; (ii) very often, an early onset transient increase of tumor edema and/or pseudo-progression which preceded the tumor size reductions; and (iii) late-onset objective responses that were achieved at a median of 287 days. To our knowledge, this is the first demonstration that the intratumoral delivery of an RNA therapeutic via extended CED in the absence of other therapeutic agents or radiation results in >3 year median PFS and >3.5 year OS in greater than one third of the efficacy population in a *R/R* HGG trial. It will be important to evaluate OT101 in difficult-to-treat IDH-wildtype, MGMT-unmethylated *R/R* HGG patients who have failed a temozolomide-based first line adjuvant therapy, as they have a dismal prognosis and are in urgent need for new and effective salvage therapies. Intratumorally administered OT101 exhibited a promising safety profile, but several CED procedure and device-related complications were identified. Therefore, risk mitigation strategies, especially preventive methods aimed at reducing the risk of infections, will be required to further improve the potential patient benefit of intratumoral OT101 therapy.

## Figures and Tables

**Table 1:** Patient Information for the modified intent-to-treat (mITT) population (*N* = 89).

Diagnosis	# (%)
AA (WHO Grade III)	27 (30.3)
GBM (WHO Grade IV)	62 (69.7)
Gender	# (%)
Female	25 (28.1)
Male	64 (71.9)
Race	# (%)
Caucasian	58 (65.2)
Asian	31 (34.8)
Black	0 (0.0)
Age (Years)	
Median (Range)	45 (19–73)
Mean ± SE	46.3 ± 1.3
KPS Score at Baseline	
Median (Range)	90 (70–100)
Mean ± SE	88 ± 1
Size of Largest Target Tumor Lesion	
2-D in cm^2^ – Median (Mean ± SE)	8.6 (9.3 ± 0.5)
3-D in cm^3^ – Median (Mean ± SE)	21.5 (27.2 ± 2.5)
OT101 Dose Cohort	# (%)
Low (2.5 mg/cycle)	40 (44.9)
High (19.8 mg/cycle)	49 (55.1)
Number of OT101 Cycles	
Median (Range)	6 (1–11)
Mean ± SE	7.0 ± 0.3
Total OT101 Dose (mg/m^2^)	
Median (Range)	22.7 (1.1–152.1)
Mean ± SE	45.2 ± 4.6
Time from last cancer therapy	
Median (Mean ± SE)	103 (248 ± 53)
Time from Diagnosis	
Median (Mean ± SE)	229 (379 ± 59)
Previous antitumor therapy	# (%)
Surgery	89 (100)
Radiotherapy	81 (91.0)
Chemotherapy	48 (53.9)
Other	13 (14.6)
Number of previous surgeries	# (%)
1	64 (71.9)
2	19 (21.3)
> 2	6 (6.7)
Number of previous radiotherapies	# (%)
0	8 (9.0)
1	73 (82.0)
2	8 (9.0)
Number of previous chemotherapies	
0	41 (46.1)
1	48 (53.9)
Number of previous other therapies	# (%)
0	76 (85.3)
1	11 (12.4)
2	2 (2.2)
Number of measurable enhancing lesions*	# (%)
1	68 (78.2)
2	14 (16.1)
3	5 (5.7)
Number of NMEL*	# (%)
0	67 (77.0)
1	12 (13.8)
2	5 (5.7)
3	3 (3.4)

* Central MRI review data were not available in two patients. NMEL: non-measurable enhancing lesions.

**Table 2:** Progression-free survival and overall survival for the modified intent-to-treat (mITT), efficacy (EP) and favorable response populations.

Parameter	mITT	mITT, <4 Cycles	Efficacy/EP 4–11 Cycles	Objective Responders *	Favorable Responders *	EP, Non-Responder	mITT, Non-Responder
**PFS (d)**	(*N* = 89)	(*N* = 12)	(*N* = 77)	(*N* = 19)	(*N* = 26)	(*N* = 51)	(*N* = 63)
Median	60	32	86	1281	1109	37	36
95% CI	39–93	32–NA	40–134	1070–NA	992–NA	35–59	35–54
**OS (d)**							
Median	399	128	432	1243	1280	240	229
95% CI	283–503	93–NA	299–788	1079–NA	1116–NA	169–361	163–318

* The three CRs were achieved in one GBM and two AA patients, the 16 PRs were achieved in four GBM and 12 AA patients. Prolonged SDs ≥6 months were achieved in five GBM and two AA patients. NA: not applicable.

**Table 3:** Univariate and multivariate analysis of PFS and OS outcome according to risk factor.

Risk Factor	PFS (Days)	χ^2^	*p*-Value	Z-Stat/*p*-Value	OS (Days)	χ^2^	*p*-Value	Z-Stat/*p*-Value
	Median (95% CI)				Median (95% CI)			
Diagnosis								
GBM (*N* = 62)	38 (35–61)	17.5	0.00003	2.4/0.018	274 (180–399)	16.1	0.00006	2.0/0.04
AA (*N* = 27)	994 (118–1423)				1136 (811–1743)			
Age (Years)								
53–73 (*N* = 30)	36 (35–86)	6.0	0.014	0.5/0.6	213 (137–341)	11.6	0.0007	0.3/0.7
19–41 (*N* = 30)	101 (59–1109)				803 (365–1243)			
KPS Score								
70–80 (*N* = 25)	40 (36–67)	6.3	0.012	0.8/0.4	162 (131–341)	16.1	0.00006	2.1/0.03
90–100 (*N* = 64)	88 (40–295)				445 (399–1069)			
OT101 Cycles								
1–3 (*N* = 12)	32 (32–NA)	16.1	0.00006	0.6/0.5	128 (93–NA)	19.5	0.00001	2.1/0.03
4–11 (*N* = 77)	86 (40–134)				432 (299–788)			
Total OT101 Dose (mg/m^2^)								
1.1–14 (*N* = 30)	36 (35–60)	2.0	0.16	2.7/0.006	222 (152–406)	3.5	0.06	2.3/0.02
53–152 (*N* = 30)	88 (40–176)				447 (402–1079)			
Dexamethasone Use								
Extensive (*N* = 25)	36 (35–86)	25.6	<0.00001	1.7/0.08	273 (152–432)	23.3	<0.00001	2.3/0.02
No or Minimal (*N* = 28)	643 (295–NA)				1172(963–NA)			
Best Overall Response (BOR)								
Non-responders— (*N* = 63)	36 (35–54)	72.6	<0.00001	16.5/<0.00001	229 (163–318)	51.6	<0.00001	5.8/<0.00001
Favorable responder—PR, CR or SD ≥ 6 months (*N* = 26)	1109 (992–NA)				1280 (1116–NA)			

Several additional variables including OT101 dose per cycle, size of target tumor lesion at baseline, time from diagnosis, time from last therapy, gender, ethnicity, hematological parameters at baseline (WBC, ALC, ANC) showed no significant predictive value for the PFS or OS outcome.

## Supplementary Materials

The following are available online at https://www.mdpi.com/2072-6694/11/12/1892/s1, Figure S1: Components of the CED System for Intratumoral OT101 Therapy. (**A**). Overview. (**B**) Implanted components. (**C**) CED system assembled. (**D**) External components, Figure S2: Time-dependent reduction of target lesion size in OT101 treated R/R adult GBM patients. These 3 GBM patients achieved an objective response by standard McDonald criteria (Table S9). In all 3 patients, review of MRI images by 2-3 independent reviewers (open circle: Reviewer 1; Closed circle: Reviewer 2; Triangle: Reviewer 3/Adjudicator) showed a time-dependent decrease of the 2-D (Panels **A**, **D**, **G**) and 3-D (Panels **B**, **E**, **H**) size of the target lesion. We also investigated the first order. kinetics of the tumor reductions in each patient by fitting a straight line to a semi-log plot of the portion of the 3-D tumor volume reduction curve that displayed maximum reduction in tumor size over the course of OT101 treatment (Panels **C**, **F** and **I**). The slope of the line represents the rate constant for tumor reduction in log10 scale, and duration times to 90% (T10: -1/Slope) and 99% (T1: -2/Slope) percent reduction of tumor volumes were calculated using the rate constant, Figure S3: Time-dependent reduction of target lesion size in OT101 treated R/R adult AA (WHO Grade III) patients. These 3 AA patients achieved an objective response by standard McDonald criteria (Table S9). In all 3 patients, review of MRI images by 2-3 independent reviewers (open circle: Reviewer 1; Closed circle: Reviewer 2; Triangle: Reviewer 3/Adjudicator) showed a time-dependent decrease of the 2-D (Panels **A**, **D**, **G**) and 3-D (Panels **B**, **E**, **H**) size of the target lesion. We also investigated the first order kinetics of the tumor reductions in each patient by fitting a straight line to a semi-log plot of the portion of the 3-D tumor volume reduction curve that displayed maximum reduction in tumor size over the course of OT101 treatment (Panels **C**, **F** and **I**). The slope of the line represents the rate constant for tumor reduction in log10 scale, and duration times to 90% (T10: -1/Slope) and 99% (T1: -2/Slope) percent reduction of tumor volumes were calculated using the rate constant, Figure S4: MRI-response of target lesion in OT101 treated R/R GBM (WHO Grade 4) patient. UPN108-138. Depicted are T1-weighted spin echo (SE) pre- and post-contrast MRI images as well as T1 SE coronal (COR) post-contrast and T2-weighted turbo spin echo (TSE) axial MRI images at baseline vs. post-treatment with OT101 on day 378 after randomization to 2.5 mg/cycle OT101 dose cohort. T1-weighted pre-contrast image at baseline exhibits a hypointense lesion in the right temporal lobe (**A**). Axial T1-weighted post-contrast axial (**B**) and coronal (**C**) images at baseline demonstrate a rimenhancing lesion stereotypical pattern of contrast enhancement and a hypo-intensity circumscribed within the enhancement that is suggestive of necrosis. The tumor and surrounding white matter within the right temporal lobe show increased signal intensity compared to a healthy brain on the T2-weighted axial MRI (**D**), consistent with extensive tumorigenic edema. Post-treatment images (**E**-**H**) show significantly decreased lesion size and edema. The BOR in this patient was a PR according to Macdonald criteria, as determined by central review of the MRI images. See Table S9 and Figure 1 for further details, Figure S5: Imaging Responses in R/R High-Grade Glioma Patients Treated with OT101 Monotherapy Who Achieved a CR or PR. (**A**) A waterfall plot depicting the maximum log10 reduction values for the tumor volumes. (**B**) A semi-log plot of the combined 3-D tumor volume reduction curve for the 19 patients. The first order kinetics of the tumor volume reduction for the entire population of the 19 objective responders is illustrated by fitting a straight line to a semi-log plot of the portion of the tumor reduction curve that displayed maximum reduction in tumor size over the course of OT101 treatment. Data points represent the individual assessments from 2-3 radiologists for each time point of MRI assessment for each of the 19 patients. The slope of the line represents the rate constant for tumor reduction in log10 scale, and duration times to 90% (T10: -1/Slope) and 99% (T1: -2/Slope) percent reduction of tumor volumes were calculated using the rate constant, Figure S6: OT101-induced Tumor Edema and Pseudo-progression prior to a Late-Onset Complete Response in R/R AA (WHO Grade 3) Patient, UPN203302. Depicted are T1-weighted spin echo (SE) (post-contrast) and T2-weighted turbo spin echo (TSE) axial MRI images obtained at baseline and at the indicated time points after randomization to the 2.5 mg/cycle dose cohort of OT101. T1-weighted axial contrast-enhanced MRI image at baseline (**A**) demonstrates an enhancing tumor in the right temporal lobe. The tumor and surrounding white matter within the right temporal lobe show increased signal intensity compared to a healthy brain on the T2-weighted axial MRI (**E**), consistent with extensive. tumorigenic edema. Follow-up imaging on day 169 demonstrates a significant increase in peripheral enhancement on the T1-weighted image (**B**). T2-weighted image demonstrates the same lesion, with notably increased edema inside the tumor and around the tumor and midline shift (**F**). These findings were not associated with clinical deterioration or need for steroid use. Subsequent images (**C**, **D** & **G**, **H**) demonstrate resolution of the enhancing lesion and edema in the absence of steroids or other cancer therapies. Figure 2 shows the T1-weighted images in higher magnification. This patient achieved a PR on day 483 and a CR on day 1838, Figure S7: Survival Outcome of HGG Patients in the Efficacy Population According to Their Best Overall Responses to OT101. (**A**) PFS outcome of the efficacy population. Favorable BOR of CR, PR or SD ≥ 6 months is associated with improved PFS in R/R HGG patients treated with OT101 monotherapy. Depicted are the PFS curves of the entire 77-patient efficacy population as well as 26 favorable responders and 51 non-responders. Patients received no other cancer therapies during the depicted PFS. See also Figure 1 and Table 2. (**B**) OS outcome of the efficacy population. Favorable BOR of CR, PR or SD ≥ 6 months is associated with improved OS in R/R HGG patients treated with OT101 monotherapy. Depicted are the OS curves of the entire 77-patient efficacy population as well as 26 favorable responders and 51 non-responders. See also Table 2, Figure S8: Relation of Histopathologic Diagnosis, Dexamethasone Use and Number of OT101 Cycles to PFS Outcome of the mITT Population. (**A**) AA patients had a significantly better outcome than GBM patients. (**B**) Patients with no or very minimal Dexamethasone use had a significantly better PFS outcome than patients with extensive Dexamethasone use. (**C**) Patients in the efficacy population (*N* = 77) receiving 4-11 cycles of OT101 had a significantly better PFS outcome than the remaining 12 patients in the mITT population who received 1-3 cycles of OT101, Figure S9: Simulated PFS Outcomes Based on Multivariate Predictive Modelling. Our investigation of the parametric PFS models resulted in a convergent solution using the loglogistic distribution function with the lowest AIC (AIC = 727, Loglikelihood of the full model = -354.3, Loglikelihood of the null intercept only model= -446.2, 85 evaluable data points, Chisq = 183.77 on 7 degrees of freedom, *p* = 3.1 x 10^-36^). The full model considered 9 parameter coefficients (7 for clinical parameters plus scale and intercept) to generate the prediction equation: Intercept = 3.85459; Age = 0.00244; (Diagnosis)GBM = -0.33467; (OT101 Cycles) 4to11 = 0.09717; Dexamethasone use = -0.23876; (KPS score) 90 to 100 = 0.09747; Total Cumulative OT101 Dose = 0.00390; (Best Overall Response) responders = 2.88321; Log(scale) = -1.33100. Depicted are simulated PFS outcomes based on multivariate predictive modelling of PFS times for AA as well as GBM patients (Simulation parameters: Dexamethasone use: None or minimal; KPS score: 90-100; Number of OT101 cycles: 4-11 (viz: efficacy population), Cumulative OT101 dose: 46 mg/m2 (=mean value for the mITT population); Age: 46 years (=mean age for mITT population) yielded 18-fold higher median PFS times for favorable responders vs. non-responders (*p* < 0.00001), Table S1: Analysis Populations of Patients Randomized to Treatment with OT101, Table S2: Overview of Adverse Events in Safety Population, Table S3: Procedure-related Serious Adverse Events, Table S4: Display of Procedure-Related Serious Adverse Events, Table S5: Incidence of OT101-Related or Possibly related Toxicity Grade 3 or 4 AE, Table S6: OT101-Related or Possibly OT101-Related Adverse Events and Serious Adverse Events Leading to Discontinuation of OT101, Table S7: Incidence of OT101-Related or - Possibly Related Adverse Events Causing Discontinuation of OT101, Table S8: Baseline Patient Characteristics for the Modified Intent-to-Treat and Efficacy Populations, Table S9: Single Agent Clinical Activity of Intratumorally Administered OT101 in R/R High Grade Glioma Patients, Table S10: Grade III/IV AE and SAE in R/R High Grade Glioma Patients Showing an Objective Response or a Prolonged Stable Disease to Intratumorally Administered OT101, Table S11: GLM-based multivariate analysis to further evaluate the predictive value of clinical parameters for favorable clinical responses to OT101 therapy, Table S12: Multivariate Analysis of Progression-Free Survival and Overall Survival, According to Risk Factor.
