# Supplementary material for: Recurrent or Refractory High-Grade Gliomas Treated by Convection-Enhanced Delivery of a TGFβ2-Targeting RNA Therapeutic: A Post-Hoc Analysis with Long-Term Follow-Up
Source: Cancers (Basel). 2019 Nov 28;11(12):1892. doi: 10.3390/cancers11121892 (PMC6966490; doi:10.3390/cancers11121892)
Supplement: Supplementary file 1 [file cancers-11-01892-s001.pdf]

Supplementary Metarials

Recurrent or Refractory High-Grade Gliomas Treated by Convection-enhanced Delivery of a TGFβ2-Targeting RNA Therapeutic: A Post-Hoc Analysis with Long-Term Follow-Up

Fatih M. Uckun, Sanjive Qazi, Larn Hwang and Vuong N. Trieu

1. Supplemental Methods

1.1. Investigational Medicinal Product and CED Drug Delivery System

Randomized groups of two OT101 dose cohorts received OT101 via a single intratumoral catheter that was implanted into target lesion. OT101 was infused intratumorally using CED (see Supplemental Methods). One treatment cycle with OT101 lasted 14 days and consisted of a 7-day administration of OT101, followed by administration of isotonic saline solution for 7 days. The port system and the intratumoral catheter for OT101 delivery were implanted 2 days prior to starting the OT101 treatments and removed after the last scheduled 7-day infusion of isotonic (0.9%) saline. OT101 dissolved in isotonic (0.9%) aqueous sodium chloride solution at a final concentration of either 10 µM or 80 µM was administered at 4 µL/min for 7 days. The total OT101 dose per cycle was 2.5 mg (10 µM group) or 19.8 mg (80 µM group). According to the clinical protocol, eligible patients assigned to an OT101 dose cohort were to be treated with OT101 for at least 8 weeks corresponding to 4 cycles of OT101 and receive a maximum of 11 treatment cycles of OT101. The investigational medicinal product (IMP) was provided to treating centers as a sterile lyophilizate in 50 mL glass vials containing 7.37 mg OT101. Prior to administration the lyophilized OT101 was reconstituted in sterile 0.9% isotonic sodium chloride solution for infusion. A concentration of either 10 µM or 80 µM of active ingredient dissolved in isotonic (0.9%) aqueous sodium chloride solution was administered. The OT101 dose delivered each cycle was either 2.5 mg or 19.8 mg. During the treatment-free intervals, blocking of the intratumoral catheter was prevented by a continuous isotonic saline infusion. Saline infusion started at a flow rate of 4 µL/min to rinse the remaining OT101 solution in the catheter. After 10 h, the flow rate was reduced to 1 µL/min for the duration of the 7-day infusion period.

The CED system depicted in Figure S1 included the following components: An implanted catheter placed intratumorally and connected subcutaneously to a port access system (consisting of port chamber and port catheter) by a connecting piece. For this purpose, a connecting piece was used between port catheter and ventricular catheter. Both catheters were barium impregnated to facilitate control of their correct placement. The port system was prefilled with isotonic (0.9%) saline solution and its postoperative position as well as the localization of the intratumoral catheter tip was controlled and documented by X-ray and CT. A porTable 2 external pump (Pegasus Vario, with study-specific configuration) (Venner Medical, Danischenhagen, Germany) ensured the delivery of the drug at the specified infusion rate of 4 µL/min. The external portable pump was connected to the port system by a special port puncture needle. The subcutaneous access system was implanted by local surgeons or neurosurgeons. The intratumoral catheter was placed by the neurosurgeons. Cranial CT (CCT) or brain MRI were performed during the preparation period for determination of the target region within the brain tumor for placement of the intratumoral catheter and calculation of the planned position of the catheter tip. A CCT scan was performed on Day -2 after placement of the intratumoral catheter to ensure correct placement of the catheter tip as well as to detect any possible procedure-related complication (e.g., hemorrhage). The local neuroradiologists, radiologists,

and their technical assistants obtained MRIs and CCTs. The described delivery system allowed treatment of eligible patients repeated OT101 infusions in an outpatient setting.

1.2. Patient Characteristics and Execution of the Clinical Trial

This was a multi-national, multi-center, open-label interventional clinical study in patients with R/R Grade III anaplastic astrocytoma (AA) or Grade IV glioblastoma (GBM). This study was conducted in three centers in Austria, one center in Georgia, eight centers in Germany, six centers in India, two centers in Israel and nine centers in Russia. The investigators were neurosurgeons or neurologists. In order to be eligible for the study, patients had to have a brain tumor (either Grade III AA or Grade IV GBM) with supratentorial localization and a measurable lesion with a maximum diameter of 4.5 cm by MRI who had no more than 2 chemotherapy regimens since diagnosis. The diagnosis of was confirmed before start of treatments. Patients had to have an expected life expectancy of ≥ 3 months and a baseline KPS score ≥70%. Patients with tumor surgery within two weeks prior to study entry were excluded as were patients receiving radiation therapy within eight weeks prior to randomization. Treatment with chemotherapy, hormone therapy, or any other therapies with established or suggested antitumor effects had to be finished 4 weeks - 6 weeks (nitrosoureas only) before randomization. No prior stereotactic radiosurgery or interstitial brachytherapy and no TGFbeta 2 (TGFb2) targeted therapy or antitumor vaccination were allowed. Patient's participation in another clinical study with investigational medication had to be completed at least 30 days prior to study entry.

98 patients (AA: 30; GBM: 68) were randomized to one of the 2 treatment arms (intent-to-treat population [ITT]) of OT101 representing 2 different dose cohorts, namely 2.5 mg/cycle (N = 48) 3 and 19.8 mg/cycle (N = 50), respectively (Table S1). 7 patients in the low dose group and 1 patient in the high dose group discontinued the study after randomization but before surgery (implantation of the catheter-port system). 90 patients (safety population/SP) underwent surgery for catheter implantation for OT101 and randomized to one of 2 dose cohorts of OT101 were evaluable for safety. One patient assigned to the low dose cohort was taken off the study after the surgical procedure but before receiving any OT101. The modified intent-to-treat (mITT) population for PFS and OS analysis included all 89 randomized patients (AA:27; GBM: 62) who had received any amount of OT101 (Table S1). One treatment cycle with OT101 lasted 14 days and consisted of a 7-day administration of OT101, followed by 7-day administration of isotonic saline solution. 20 patients, including 11 in the 2.5 mg dose cohort and 9 in the 19.8 mg dose cohort participated in prolonged follow-up (PFU) evaluations after completion of the core study.

The administered concentration of OT101 was either 10 μM (Dose cohort: 2.5 mg/cycle) or 80 μM (Dose cohort: 19.8 mg/cycle). Patients were to be treated with OT101 for at least 8 weeks corresponding to 4 cycles of OT101 and receive a maximum of 11 treatment cycles of OT101. Of the 89 patients randomized to OT101 treatments, only 77 (efficacy population) received the intended minimum number of 4 OT101 treatment cycles (Table S1). No other cancer treatments, standard or experimental (including but not limited to radiation therapy, chemotherapy, immunotherapy) were administered unless the patient experienced progression of disease.

OT101 was administered via continuous infusion over 7 days to 89 adults (62 GBM and 27 AA patients) with R/R HGG via intracranial delivery with an intratumoral catheter using a CED system. The intended minimum number of the 7-day OT101 cycles was 4 and the maximum allowed number of 7-day OT101 cycles was 11. Activity and efficacy analyses were performed for the mITT population (i.e., all randomized 89 patients who were treated with OT101) and for the efficacy population (i.e., all 77 patients who received a minimum of 4 cycles of OT101). The mITT population included 25 females and 64 males at a median age of 45 (Range: 19–73; Mean ± SE = 46.3 ± 1.3) years with a median baseline KPS score of 90 (Range 70–100; Mean ± SE: 87.6 ± 0.9). Patient characteristics are shown in Table 1. 58 patients were Caucasian whereas 31 were Asian. 62 patients had GBM and 27 had AA. 40 patients were treated at the low dose level (10 μM concentration in the infusate; 2.5 mg/cycle) and 49 patients were treated at the high dose level (80 μM concentration in the infusate; 19.8 mg/cycle) of OT101. The mean size of the target lesion for the mITT population was 9.3±0.6 cm^2 for 2-D surface

area measurements 4 and 27.1±2.5 cm^3 for 3-D volume measurements. 68 patients (78.2%) had a single measurable contrast-enhancing lesion and non-measurable contrast-enhancing lesions were reported only in 20 (22.5%) patients (Table 2). The median time from first diagnosis to randomization was 229 (Mean ± SE: 379 ± 59) days and the median time from last cancer therapy to randomization was 103 (Mean ± SE: 248 ± 53) days. Patients received 7.0±0.3 (Range: 1–11; Median: 6) cycles of OT101 at an average (Mean ± SE) total cumulative dose of 45.2 ± 4.6 (Median: 22.7, Range: 1.1–152.1) mg/m^2.

2. Study Approval

No human subjects were involved in this post-hoc analysis of the Phase IIB study G004. The primary Phase IIB study was registered with ClinicalTrials.gov (Study No. NCT00431561). The primary study NCT00431561 was performed in compliance with all applicable regional and national regulations and with approval from independent ethics committees and Institutional Review Boards of the participating institutions. Each patient provided a written informed consent.

3. Safety and Efficacy Measurements

Safety analyses were performed for all 90 patients in the safety population (SP). AE terms were coded using the Medical Dictionary for Regulatory Activities (MedDRA). Safety laboratory analyses (hematology, biochemistry, and urine analysis) were performed by the respective local hospital laboratories of each involved site. In India, a central laboratory (SIRO Prolego, Mumbai, India) as well as the local hospital laboratories were used for safety laboratory analyses.

Activity and efficacy analyses were performed for the mITT population (i.e., all randomized 89 patients who were treated with OT101) and for the primary efficacy population (i.e., all 77 patients who received the intended minimum of 4 cycles of OT101). For immediate decisionmaking during the course of the study, the local neuroradiologists evaluated patients' local MRIs according to study-specific procedures, filed in the TMF. For a standardized response assessment for the study analysis, an independent Central MRI Reading (CMRIR) was performed by a specialized central reading institute (Timaq Medical Imaging Inc, Zurich, Switzerland). Central reading was conducted by two independent neuroradiologists with an additional adjudicator for cases of predefined discrepancies in the reports of the two readers. 5 The axial T2- and T1-weighted sequences were performed in identical slice positions to ensure comparability. The coronal scans were oriented parallel to the dorsal contour of the brain stem at the level of the pons. The sagittal T1-weighted 3D sequence covered the whole brain. Before the IV injection of contrast medium (CM), the acquisition sequence included T2 axial, native (Turbo spin echo/TSE or fast spin echo/FSE, slice thickness: 6 mm, Gap: 0.6 mm, TE/Echo time: 80-120 msec) and T1 axial native (spin echo/SE, not TSE, slice thickness: 6 mm, Gap: 0.6 mm, TE/ Echo time: 80–120 msec) images. One minute after intravenous injection of Gadolinium-based CM (0.1 mmol/kg body weight), the acquisition sequence included T1 axial + CM (SE, not TSE; slice thickness: 6 mm; gap: 0.6 mm, TE: 12–20 msec), T1 coronal + CM (SE, not TSE; slice thickness: 6 mm; gap: 1.2–1.8 mm, TE: 12-20 msec) and T1-3D gradient echo, sagittal + CM (to cover the whole brain, slice thickness: maximum 1.5 mm) images.

Best overall response (BOR) was defined as the best response (i.e., CR, PR or SD) observed from the start of treatment until disease progression. For determining the treatment response of individual patients to OT101, standard MacDonald criteria were used. Complete Response (CR) was defined as the disappearance of all enhancing tumor on consecutive MRIs (at least one month apart), off steroids. Partial Response (PR) was defined as > 50% reduction in size of enhancing tumor on consecutive MRIs (at least one month apart), steroids stable or reduced. Similar criteria for CR and PR were applied to collect pilot data according to Modified Macdonald Criteria which additionally took into consideration the relevance of edema, necrosis, bleeding to be taken into consideration. CR and PR were confirmed by two consecutive observations not less than four weeks apart.

Duration of objective response was defined as the interval from the onset of CR or PR to SD, PD or death due to any cause, whichever occurred first. Patients who do not progress or die were censored at the last tumor assessment date. Time to progression (TTP) was calculated for all patients from the date of randomization to the date of the first documented tumor progression. Patients who

were switched to another anti-tumor therapy were assumed progressed at the time of switch even if the progression was not documented by MRI assessments. Patients who remained alive without PD were censored at last follow-up. If the patient had not shown clinical signs of progression, continuation of study treatment was allowed in case of tumor progression according to MRI assessment within the first three months. 6 Overall survival (OS) was the time from the date of randomization to time of death. Surviving patients were censored at their last follow-up. Progression-free survival (PFS) was the time from randomization to documentation of PD or death. Patients who remained alive without PD were censored at last follow-up. Standard definitions were used for time to progression and duration of objective response.

4. Statistical Analyses

The distribution of time-to-event survival end points on the OS and PFS curves were estimated by the Kaplan-Meier method [1]. Differences between groups were evaluated by log-rank statistics. DoR and median DoR were also estimated by the Kaplan-Meier method and compared using the log-rank test. The analyses were performed using JMP software (version 10.02, SAS Institute, Inc, Cary, NC), and R software, version 3.5.2 (R Foundation for Statistical Computing) loaded with statistical packages for survival analysis (survMisc_0.5.5; survival_2.44-1.1 and survminer_0.4.4) with default settings). For the patients who had a CR, PR or SD ≥6 months as their BOR, Waterfall plots were used to represent the maximum percentage or $\log_{10}$ change in MRI-based tumor volume of the target lesion relative to measurements taken at baseline. Vertical bars on these plots measured maximum percent reduction in tumor volumes or in terms of maximum $\log_{10}$ reduction in tumor volumes following OT101 treatment. To test whether a fixed fraction of the tumor cells is killed regardless of the tumor size, we investigated the first order kinetics of the tumor reductions in each of the objective responder patients (viz., patients with a CR or PR as their BOR) by fitting a straight line to a semi-log plot of the portion of the tumor growth curve that displayed maximum reduction in tumor size over the course of OT101 treatment. The slope of the line represents the rate constant for tumor reduction in $\log_{10}$ scale, and times to 90% (T_{10}: -1/Slope) and 99% (T_1: - 2/Slope) percent reduction of tumor volumes were calculated using the rate constant. For the patients who achieved a CR or PR as their BOR after OT101 treatments, the onset of PR and/or CR, duration of CR/PR, end of OR and onset of PD were charted utilizing Swimmer plots. Significance of continuous predictor variables for OS were determined utilizing a two arm Kaplan Meier analysis whereby OS the top third of the patients for measurements for the variable being tested was compared to the bottom third of the patients with lowest dimensions for the variable being tested. Categorical variables were also tested to predict survival outcome. These models tested each of the clinical parameters separately.

We explored effects of several clinical parameters to predict BOR as well as PFS/OS outcome in a multivariate setting. Multivariate models took into account cross correlations between the prediction variables to identify independent predictors of survival. Two approaches were utilized: (i) Generalized Linear Model was fitted to a binomial response model to determine the best predictors for proportion of responders relative to non-responders); and (ii) The parametric survival model was utilized to fit the time to death using multivariate linear regression for prediction of improved PFS/OS influenced by the clinical parameters. The best fit parametric survival model was chosen utilizing both Akaike information criterion (AIC) and Bayesian information criterion (BIC) from assessing from assessing a number of distributions of the survival probabilities (JMP 10.02, SAS, Cary NC or R version 3.5.2). Generalized Linear Model was fitted to the binomial response model to determine the best predictors for proportion of responders relative to non-responders using the "stats" package deployed in Rstudio Version 1.1.463 front end running in the R version 3.5.2 programming environment. The favorable responders were defined as patients who achieved CR, PR or SD≥ 6 months (N=26). The response variable was the proportion of responders and the predictor variables were the clinical predictor parameters. Regression models were constructed utilizing the Logit link function (effect sizes = Ln(P/1-P); P is the proportion of an event) that linked expected value of the response to the linear predictor of explanatory variables. The distribution of the proportions were assumed to be binomial as each patient can be either a responder or non-responder. The fitted

parameter values were divided by standard errors determined from the residuals of the fit resulting in the calculation of the Z-statistic (R version 3.5.2) that was used for P-value estimation [2]. Univariate and mutivariate models were fitted to the clinical parameters. Pvalues of less than 0.05 for the predictors were considered significant. Significant effect sizes that utilized chi-square distribution of errors were calculated utilizing the maximum likelihood estimation (MLE) with the use of Firth's bias-adjusted estimates to minimize the effects of separability (predictors variables that perfectly separate into response variable), small sample sizes, and bias of the parameter estimates (JMP 10.02, SAS, Cary NC). To assess the suitability of the fitted model, the studentized Pearson residuals versus predictor variables were plotted to visualize the distribution of the residuals. Maximized log-likelihood functions were calculated to determine significance of the model fit to the data, whereby these functions were used to calculate statistical deviance of generalized linear model the null deviance (intercept only model) from the residual deviance of the full model that includes the clinical parameters. The fitted parameter values were divided by standard errors determined from the residuals of the fit resulting in the calculation of the Z-statistic that was used for P-value estimation [2]. Univariate and mutivariate models were fitted to the clinical parameters. P-values of less than 0.05 for the predictors were considered significant.

All models consisted of a number of predictors for proportion of responders in the model. To visualize the relationships of these predictors to the response, prediction profiles were plotted from the best fit parameters of the GLM or PFS/OS models. The initial model contained 12 predictor variables. Time from last date of chemotherapy/radiation therapy to date of randomization, time from first diagnosis to date of randomization and the hematological measurements (ALC, ANC and WBC) were removed from the model as they were not significant predictors of PFS/OS. The second approach also screened all measured potential predictors of improved survival times using a parametric regression platform that fits a linear regression model accounting for survival (PFS or OS) probabilities that include censoring. The parametric survival platform fitted the time to death of each patient using linear regression models that can calculate both location and scale effects (JMP 10.02, SAS, Cary NC). Residual quantile plots were generated to visualize distribution of the estimated errors and to identify outliers. P-values estimated using the c2 distribution for the parameters of the model of less than 0.05 were deemed significant. We investigated 12 clinical predictor variables by initially utilizing a 2-factor parametric regression model and then expanding the model to up to 7 factors limited by the number of degrees of freedom in the full model. Patient demographic information (age, sex, ethnicity), hematological measurements (WBC, ALC, ANC) and treatment parameters (OT101 dose, number of cycles, steroid use, previous therapies prior to OT101 treatment) were investigated for their effect on PFS/OS in conjunction with the type of cancer (GBM or AA). At first a 2-factor model was constructed the included the effect of cancer subtype and one other predictor variable. The hematological parameters, gender and ethnicity were not significant in the 2-factor models. Then a multiple regression model that included up to 7 factors was used to determine variables whose effect was mitigated in a multivariate setting. Time from last date of chemotherapy/radiation therapy to date of randomization, time from first diagnosis to date of randomization and the hematological measurements (ALC, ANC and WBC) were removed from the model as they were not significant predictors of PFS or OS.

Significance of continuous predictor variables for OS/PFS were further determined utilizing a two arm Kaplan Meier analysis whereby OS/PFS the top third of the patients for measurements for the variable being tested was compared to the bottom third of the patients with lowest dimensions for the variable being tested. Categorical variables were also tested to predict survival outcome. These models tested each of the clinical parameters separately. Survival curves were visualized using the survminer graphing package (Drawing Survival Curves using 'ggplot2'. R package version 0.4.4; https://CRAN.R-project.org/package=survminer). Parametric regression methods for time to event survival analysis were employed to identify confounding and independent predictor variables from the most significant effects charaterized in the univariate analyses (ggplot2 v3.1.0, flexsurv v1.1.1, survMisc v0.5.5, survival v2.44-1.1 and survminer v0.4.4 statistical packages were deployed using Rstudio Version 1.1.463 front end running in the R version 3.5.2 programming environment). OS/PFS

response with censoring was predicted by continuous or categorical factors utilizing all the OT101-treated patients (*N* = 89). Proportions of patients surviving were fitted using 6 distributions (weibull, gamma, exponential, log-logistic, log-normal, or gompertz). The model with the lowest Akaike information criterion (AIC) score was used to calculate effect sizes and p-values to identify significant parameters in the model that included an intercept. Optimization of the parameters in the model was determined by maximizing the log-likelihood probability density functions in order to obtain standard errors for each of the parameters tested in the model. The significance of the parameter was assessed by dividing the parmeter value by the standard error value to obtain the Z-statistic for which the p-value was determined from the normal distribution of errors [3,4]. The parameters in the model were utilized to generate prediction equations to investigate the effects of the most significant independent predictors on median survival times visualized using the graphing tools in R (ggplot2 package).

References

1. Kaplan, E.L.; Meier, P. Nonparametric estimation from incomplete observations. J Am Stat Assoc. **1958**, *53*, 457–81.
2. Fox,J.; Weisberg, S. 2018. An R companion to applied regression, 3rd Edition, SAGE Publications, Publisher Location, New Bury Park, CA, USA, 2018; pp. 1-607. ISBN 978-1-5443-3647-3.
3. Jackson, C.H. Flexsurv: A Platform for Parametric Survival Modeling in R, J Stat Softw. **2016**, *70*. pii: i08. doi: 10.18637/jss.v070.i08;
4. Terry, M.; Therneau, P.; Grambsch, M. Modeling Survival Data: Extending the Cox Model. Springer, New York. USA,2000, pp. 1-287. ISBN 0-387-98784-3.

5. Supplemental Data

Adverse Events and Reactions Associated with Convection-Enhanced Delivery of OT101 via an Intratumoral Catheter. Safety analyses were performed for all 90 patients in the safety population (SP), which included all randomized patients who underwent the catheter implantation surgery or OT101 treatments (see Table S2–S8). Most of the AE or SAE were not related to OT101 but rather to the neurological disorders (e.g., increased intracranial pressure or brain edema) associated with the underlying HGG. In most cases, the investigators regarded progression of the primary disease as the cause for the AEs and SAEs. As detailed in Table S3 and S4, 28 patients (31.1%) experienced surgical procedure-related (possibly, likely or definetely related) SAE associated with the implantation of the intratumoral catheter and use of the CED system. Intratumorally administered OT101 exhibited a promising safety profile, as documented in Tables S5–S7 (see Supplementary Material). Only 10 (11.1%) experienced OT101-related/possibly related Grade 3 or 4 AE (Table S5), and only 2 patients (2.2%) experienced OT101-related SAE leading to discontinuation of OT101 (Table S6,S7).

Relation of Histopathologic Diagnosis, Performance Status, Age, Dexamethasone Use to PFS and OS Outcomes of R/R HGG Patients Treated with Intratumorally Delivered OT101. Cancer subtype was a significant predictor of PFS and OS. The median PFS for 27 patients with AA within the mITT population was 994 (95% CI: 118 - 1423) days compared to 62 patients with GBM that exhibited a median PFS of only 38 (95% CI: 35 - 61) days. This difference in PFS outcome was highly statistically significant (Log-rank c2 = 17.5, *p*-Value < 0.0001) (Table 3, Table S12). Likewise, the median OS for 27 patients with AA within the mITT population was 1136 (95% CI: 811 - 1743) days, whereas the median OS for 62 patients with GBM was only 274 (95% CI: 180 - 399) days (Table 3, Figure S8). This difference in OS outcome was also highly statistically significant (Log-rank c2 = 16.1, *p*-Value < 0.0001). Further, older patients within the mITT population had worse PFS and OS outcomes than younger patients. The median PFS of the top one third (*N* = 30) oldest patients (age range: 53–73 years) was only 36 (95% CI: 35 - 86) days, whereas the median PFS for the top one third youngest patients (age range: 19–41 years) (*N* = 30) was 101 (95% CI: 59 - 1109) days (Table 3). This PFS outcome difference was statistically significant (Log-rank c2 = 6.0, *p*-Value = 0.014). The median OS for the top one third oldest patients (age range: 53–73 years) was 213 (95% CI: 137 - 341) days, *N* = 30, whereas the median OS for the top one third youngest patients (age range: 19–41 years) was 803 (95% CI: 365 - 1243) days, *N* = 30, Log-

rank c^2 = 11.6, *p*-Value < 0.001) (Table 3), indicating that patient age was a strong predictor of survival outcome after OT101 therapy.

The median PFS for 25 patients with baseline KPS scores of 70–80 was only 40 (95% CI: 36 - 67) days compared to the median PFS of 88 (95% CI: 40 - 295) days for 64 patients with baseline KPS cores of 90–100. The difference in PFS outcome for these 2 groups was statistically significant (Log-rank c^2 = 6.3, *p*-Value = 0.01). Similarly, the median OS for the 25 patients within the mITT population who had baseline KPS scores of 70-80 was only 162 (95% CI: 131 - 341) days. This survival outcome was significantly worse than the survival outcome of the remaining 64 patients of the mITT population with KPS scores of 90-100 whose median OS was 445 (95% CI: 399 - 1069) days (Log-rank c^2 = 16.1, *p*-Value < 0.0001). Hence the performance status of the patients emerged as a strong predictor of their survival outcome after OT101 therapy (Figure S8, Table 3).

Notably, patients who had received either no dexamethasone or very limited amounts of dexamethasone for treatment of specific AE only had much better PFS and OS outcomes than the remaining patients who had more extensive dexamethasone use (Table 3, Figure S8). The difference in PFS outcome for these 2 groups was highly statistically significant (Log-rank c^2 = 23.5, *p*-Value < 0.0001). Similarly, the median OS of the 27 patients with limited dexamethasone use was 1172 (95% CI: 963 - NA) days which was significantly better than the median OS of 273 (95% CI: 152 - 432) days for the 25 patients with extensive dexamethasone use days (Log Rank Chi Square = 21.5, P-value <0.0001) (Table 3, Figure S8).

The median OS for the 30 patients with largest treated target lesions (30-125 cm^3) representing the top third of patients within the mITT population relative to the 3D tumor volume of their treated target lesion was 274 (95% CI: 163 - 399) days representing a trend (Log-rank c^2 = 3.37, *p*-Value = 0.07) toward a worse outcome when compared to the median OS of 474 (95% CI: 402 - 1116) days for the 30 patients representing the bottom third of at risk patients relative to the 3D tumor volume (1.3-16.7 cm^3) of their treated target lesion.

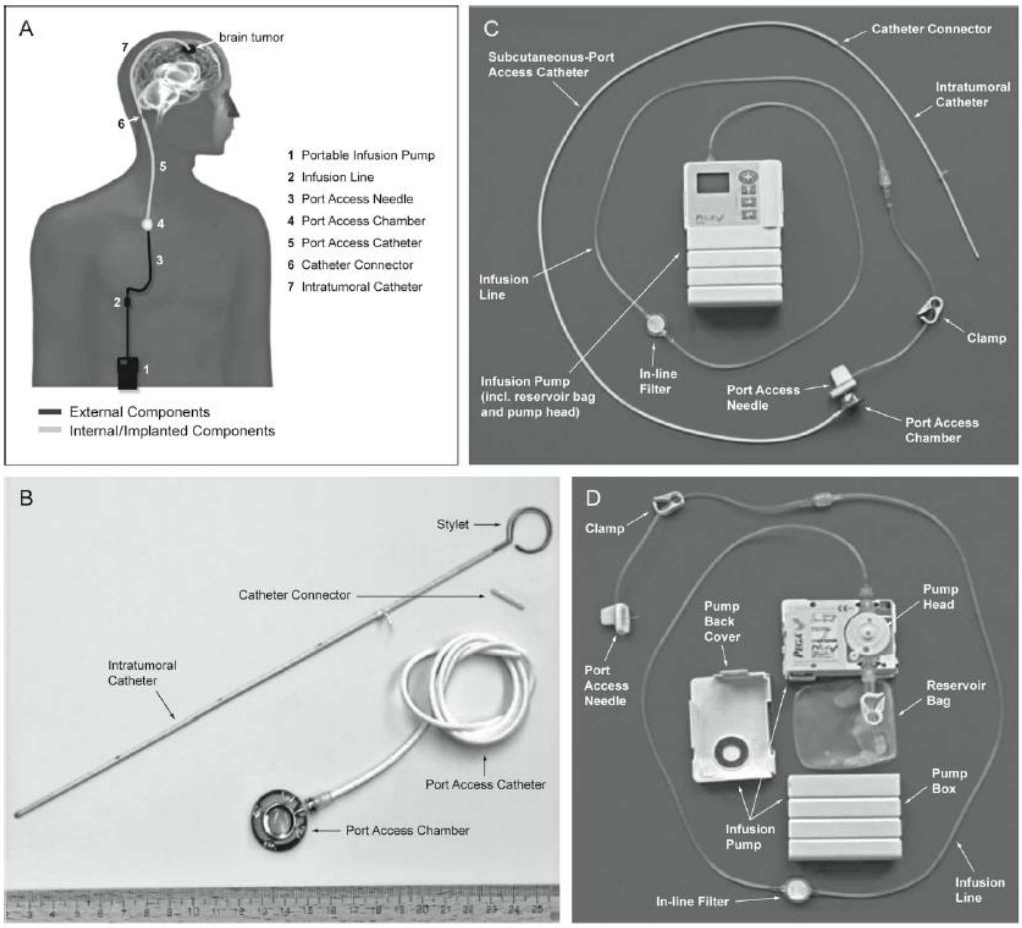

Figure S1. Components of the CED System for Intratumoral OT101 Therapy. (**A**). Overview. (**B**) Implanted components. (**C**) CED system assembled. (**D**) External components.

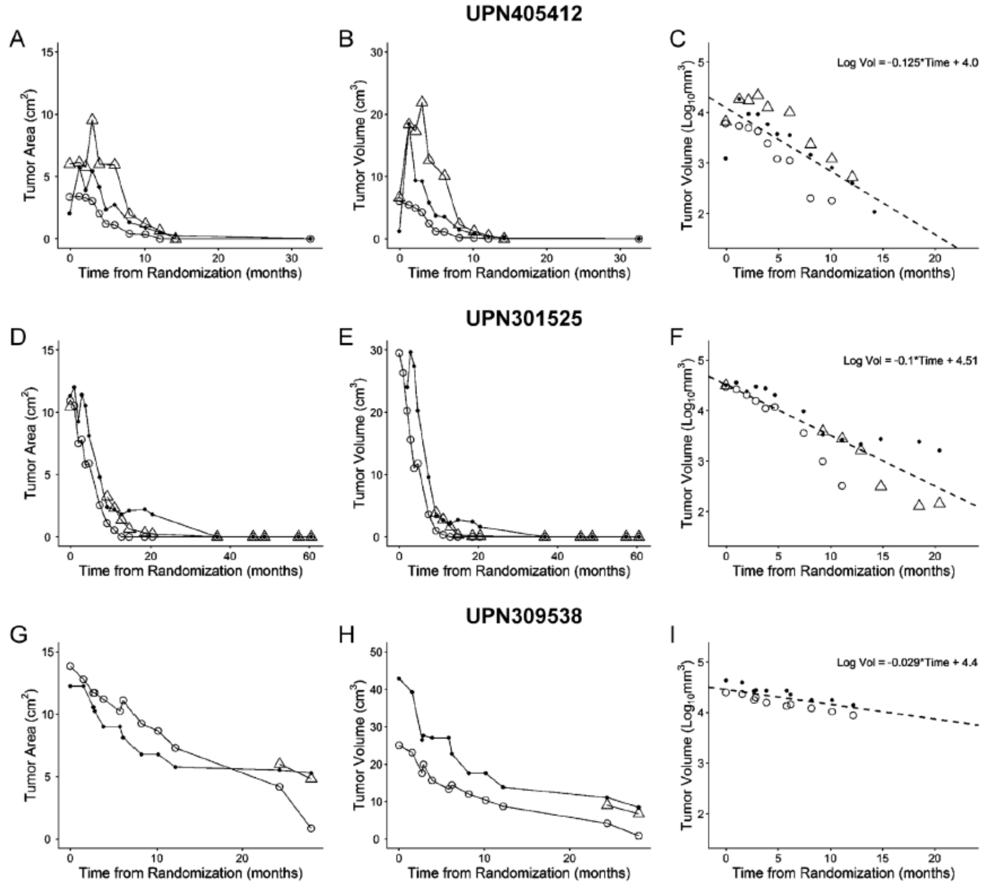

Figure S2: Time-dependent reduction of target lesion size in OT101 treated R/R adult GBM patients. These 3 GBM patients achieved an objective response by standard McDonald criteria (Table S9). In all 3 patients, review of MRI images by 2-3 independent reviewers (open circle: Reviewer 1; Closed cirde: Reviewer 2; Triangle: Reviewer 3/Adjudicator) showed a time-dependent decrease of the 2-D (Panels **A, D, G**) and 3-D (Panels **B, E, H**) size of the target lesion. We also investigated the first order. kinetics of the tumor reductions in each patient by fitting a straight line to a semi-log plot of the portion of the 3-D tumor volume reduction curve that displayed maximum reduction in tumor size over the course of OT101 treatment (Panels **C, F** and **I**). The slope of the line represents the rate constant for tumor reduction in log_{10} scale, and duration times to 90% (T_{10}: -1/Slope) and 99% (T_1: -2/Slope) percent reduction of tumor volumes were calculated using the rate constant.

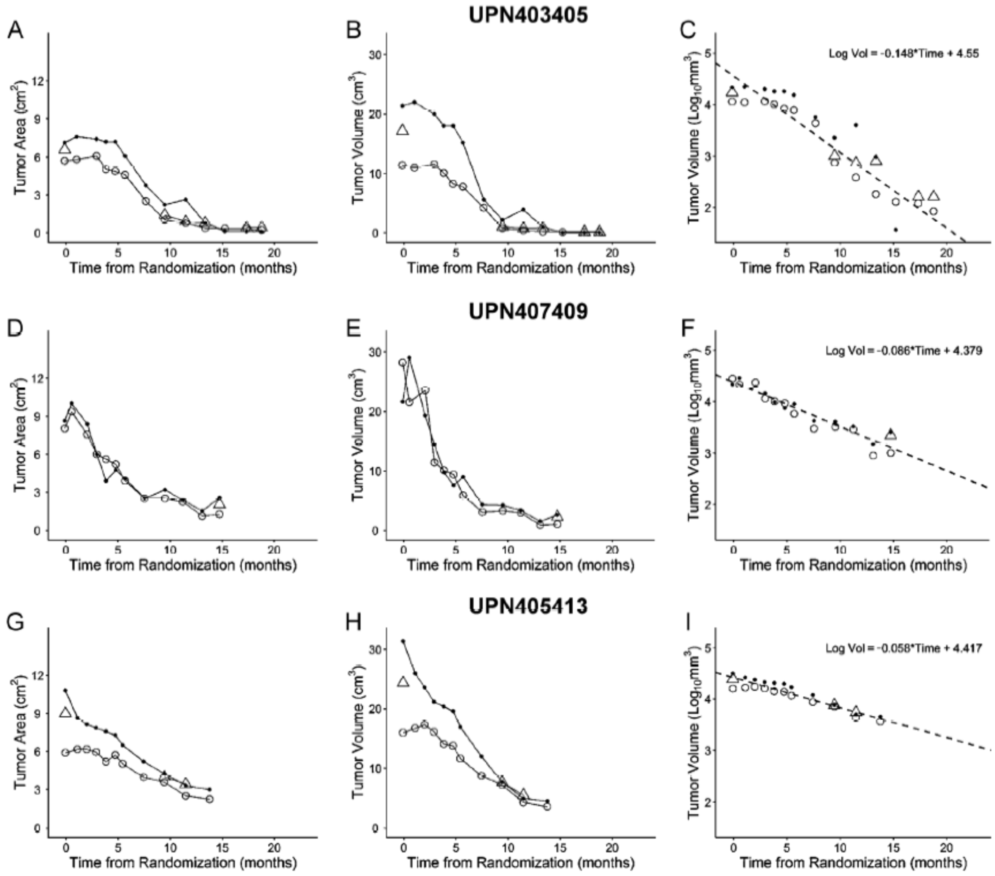

Figure S3. Time-dependent reduction of target lesion size in OT101 treated R/R adult AA (WHO Grade III) patients. These 3 AA patients achieved an objective response by standard McDonald criteria (Table S9). In all 3 patients, review of MRI images by 2-3 independent reviewers (open circle: Reviewer 1; Closed circle: Reviewer 2; Triangle: Reviewer 3/Adjudicator) showed a time-dependent decrease of the 2-D (Panels **A, D, G**) and 3-D (Panels **B, E, H**) size of the target lesion. We also investigated the first order kinetics of the tumor reductions in each patient by fitting a straight line to a semi-log plot of the portion of the 3-D tumor volume reduction curve that displayed maximum reduction in tumor size over the course of OT101 treatment (Panels **C, F** and **I**). The slope of the line represents the rate constant for tumor reduction in $\log_{10}$ scale, and duration times to 90% (T_{10}: -1/Slope) and 99% (T_1: -2/Slope) percent reduction of tumor volumes were calculated using the rate constant.

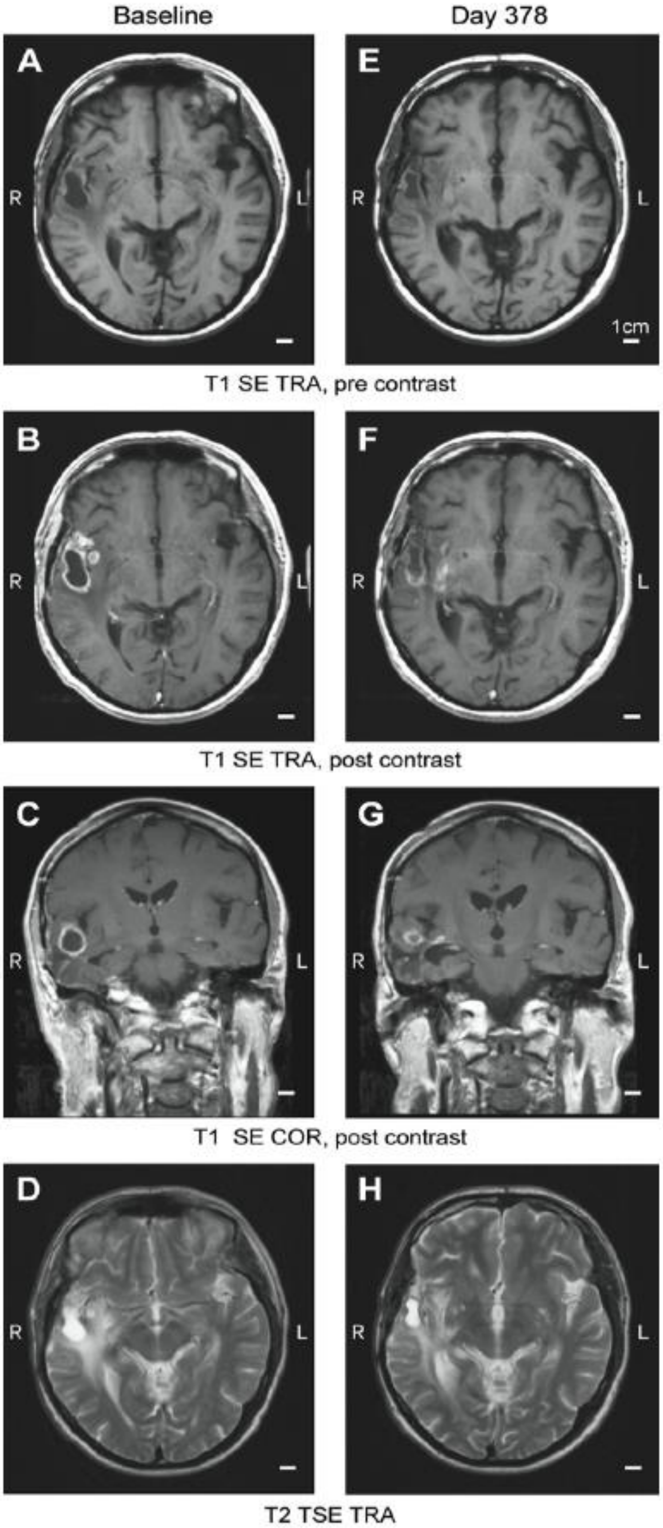

Figure S4. MRI-response of target lesion in OT101 treated R/R GBM (WHO Grade 4) patient. UPN108-138. Depicted are T1-weighted spin echo (SE) pre- and post-contrast MRI images as well as T1 SE coronal (COR) post-contrast and T2-weighted turbo spin echo (TSE) axial MRI images at baseline vs. post-treatment with OT101 on day 378 after randomization to 2.5 mg/cycle OT101 dose cohort. T1-weighted pre-contrast image at baseline exhibits a hypointense lesion in the right temporal lobe (**A**). Axial T1-weighted post-contrast axial (**B**) and coronal (**C**) images at baseline demonstrate a rimenhancing lesion stereotypical pattern of contrast enhancement and a hypo-intensity circumscribed within the enhancement that is suggestive of necrosis. The tumor and surrounding white matter within the right temporal lobe show increased signal intensity compared to a healthy

brain on the T2-weighted axial MRI (**D**), consistent with extensive tumorigenic edema. Post-treatment images (**E-H**) show significantly decreased lesion size and edema. The BOR in this patient was a PR according to Macdonald criteria, as determined by central review of the MRI images. See Table S9 and Figure 1 for further details.

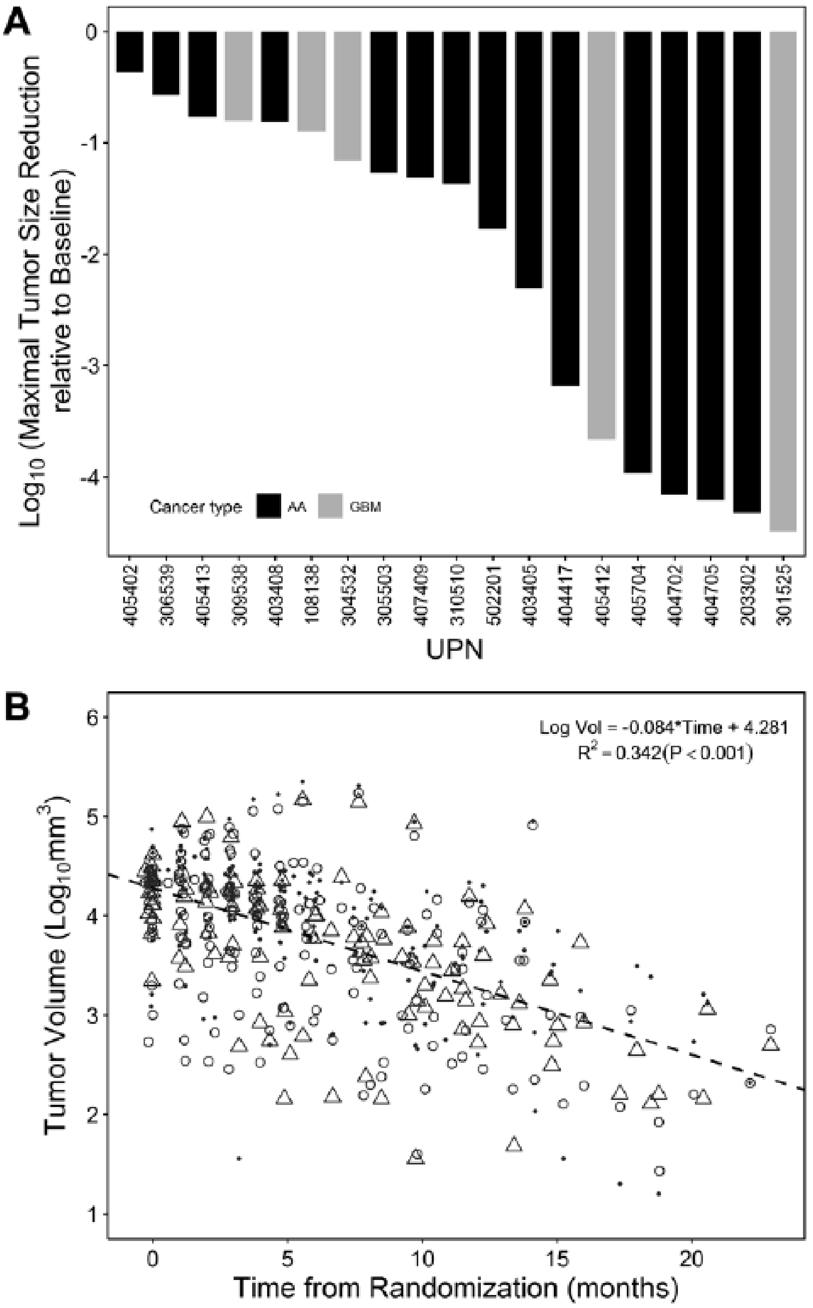

Figure S5. Imaging Responses in R/R High-Grade Glioma Patients Treated with OT101 Monotherapy Who Achieved a CR or PR. (**A**) A waterfall plot depicting the maximum log10 reduction values for the tumor volumes. (**B**) A semi-log plot of the combined 3-D tumor volume reduction curve for the 19 patients. The first order kinetics of the tumor volume reduction for the entire population of the 19 objective responders is illustrated by fitting a straight line to a semi-log plot of the portion of the tumor reduction curve that displayed maximum reduction in tumor size over the course of OT101 treatment. Data points represent the individual assessments from 2-3 radiologists for each time point

of MRI assessment for each of the 19 patients. The slope of the line represents the rate constant for tumor reduction in $\log_{10}$ scale, and duration times to 90% (T_{10}: -1/Slope) and 99% (T_1: -2/Slope) percent reduction of tumor volumes were calculated using the rate constant.

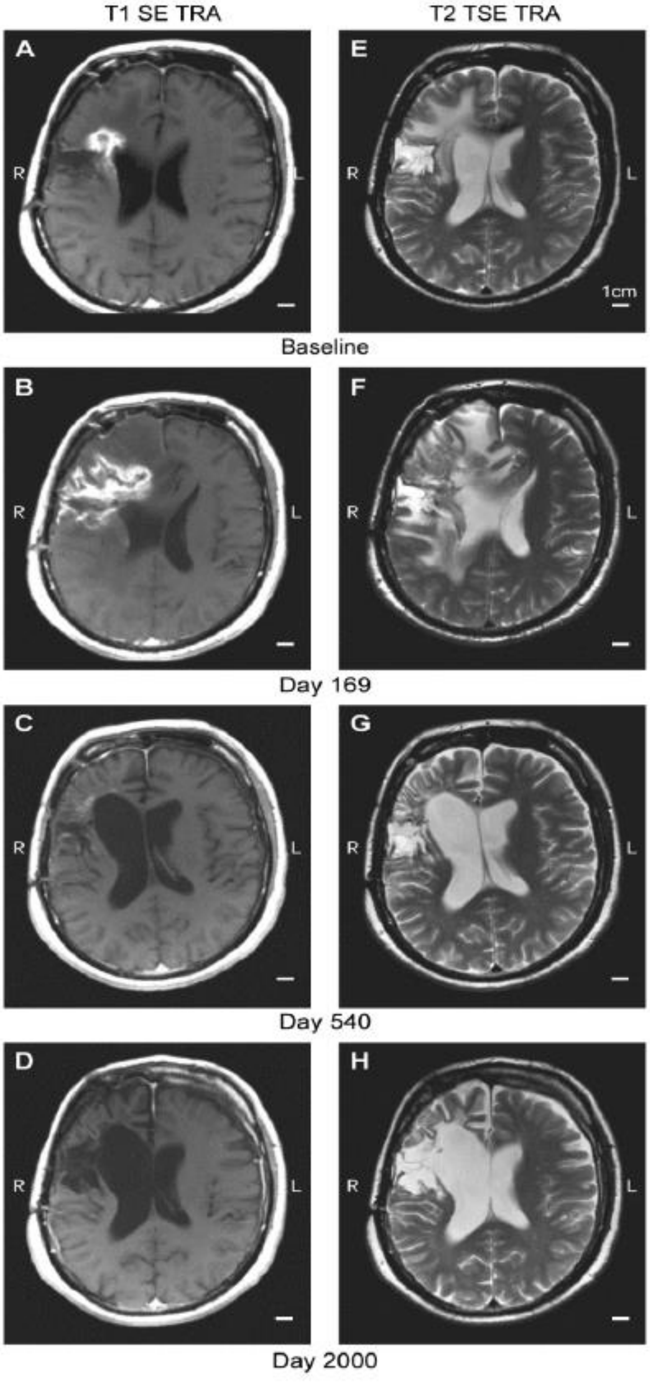

Figure S6. OT101-induced Tumor Edema and Pseudo-progression prior to a Late-Onset Complete Response in R/R AA (WHO Grade 3) Patient, UPN203302. Depicted are T1-weighted spin echo (SE) (post-contrast) and T2-weighted turbo spin echo (TSE) axial MRI images obtained at baseline and at the indicated time points after randomization to the 2.5 mg/cycle dose cohort of OT101. T1-weighted axial contrast-enhanced MRI image at baseline (**A**) demonstrates an enhancing tumor in the right temporal lobe. The tumor and surrounding white matter within the right temporal lobe show increased signal intensity compared to a healthy brain on the T2-weighted axial MRI (**E**), consistent

with extensive. tumorigenic edema. Follow-up imaging on day 169 demonstrates a significant increase in peripheral enhancement on the T1-weighted image (**B**). T2-weighted image demonstrates the same lesion, with notably increased edema inside the tumor and around the tumor and midline shift (**F**). These findings were not associated with clinical deterioration or need for steroid use. Subsequent images (**C, D & G, H**) demonstrate resolution of the enhancing lesion and edema in the absence of steroids or other cancer therapies. Figure 2 shows the T1-weighted images in higher magnification. This patient achieved a PR on day 483 and a CR on day 1838.

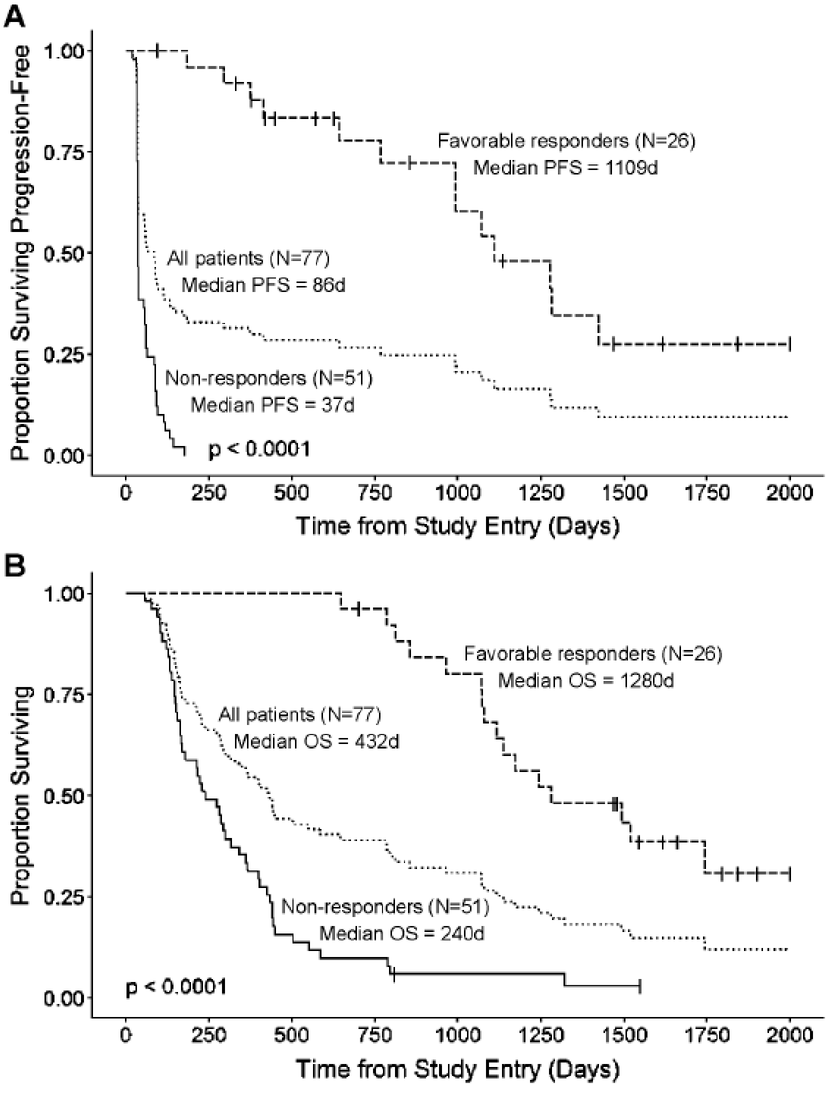

Figure S7. Survival Outcome of HGG Patients in the Efficacy Population According to Their Best Overall Responses to OT101. (**A**) PFS outcome of the efficacy population. Favorable BOR of CR, PR or SD ≥ 6 months is associated with improved PFS in R/R HGG patients treated with OT101 monotherapy. Depicted are the PFS curves of the entire 77-patient efficacy population as well as 26 favorable responders and 51 non-responders. Patients received no other cancer therapies during the depicted PFS. See also Figure 1 and Table 2. (**B**) OS outcome of the efficacy population. Favorable BOR of CR, PR or SD ≥ 6 months is associated with improved OS in R/R HGG patients treated with OT101 monotherapy. Depicted are the OS curves of the entire 77-patient efficacy population as well as 26 favorable responders and 51 non-responders. See also Table 2.

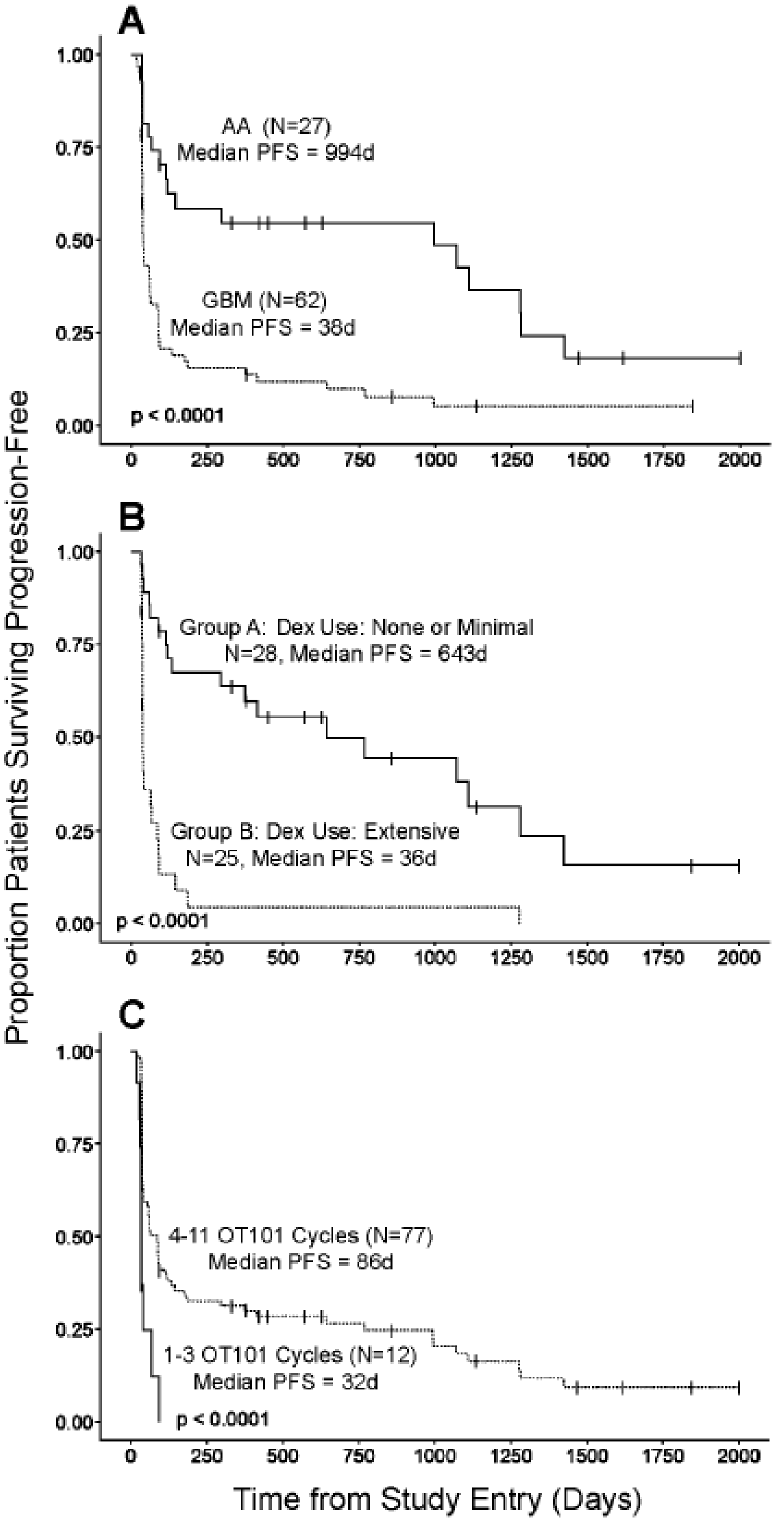

Figure S8. Relation of Histopathologic Diagnosis, Dexamethasone Use and Number of OT101 Cycles to PFS Outcome of the mITT Population. (**A**) AA patients had a significantly better outcome than GBM patients. (**B**) Patients with no or very minimal Dexamethasone use had a significantly better PFS outcome than patients with extensive Dexamethasone use. (**C**) Patients in the efficacy population (*N* = 77) receiving 4-11 cycles of OT101 had a significantly better PFS outcome than the remaining 12 patients in the mITT population who received 1-3 cycles of OT101.

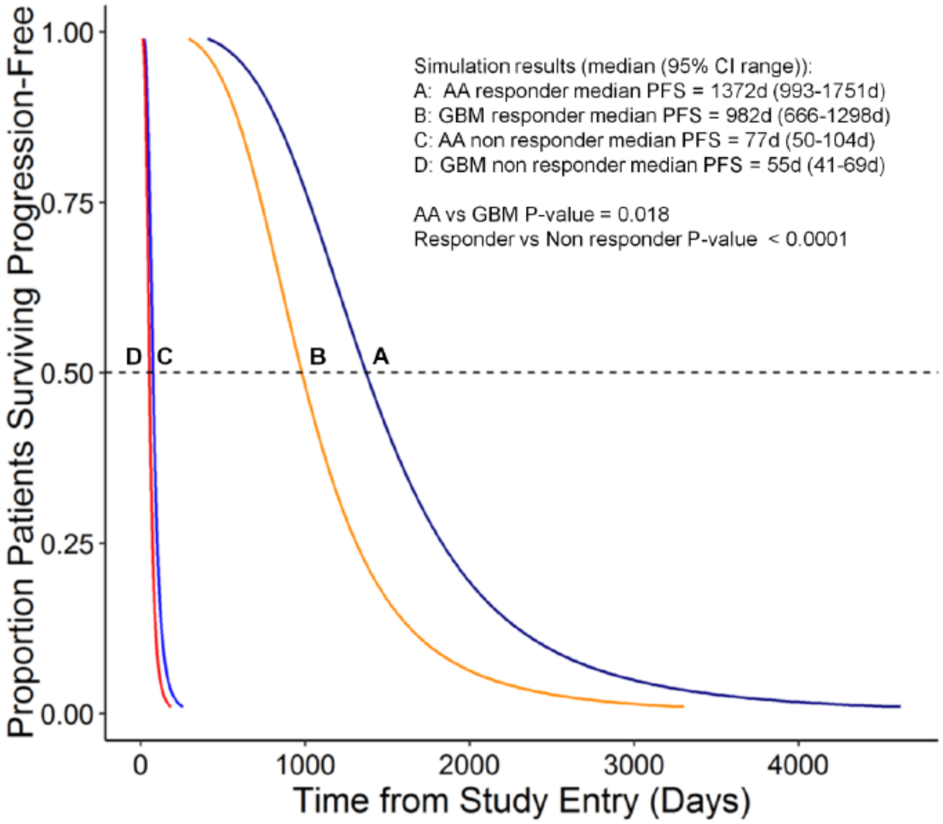

Figure S9. Simulated PFS Outcomes Based on Multivariate Predictive Modelling. Our investigation of the parametric PFS models resulted in a convergent solution using the loglogistic distribution function with the lowest AIC (AIC = 727, Loglikelihood of the full model = -354.3, Loglikelihood of the null intercept only model= -446.2, 85 evaluable data points, Chisq = 183.77 on 7 degrees of freedom, $p = 3.1 \times 10^{-36}$). The full model considered 9 parameter coefficients (7 for clinical parameters plus scale and intercept) to generate the prediction equation: Intercept = 3.85459; Age = 0.00244; (Diagnosis)GBM = -0.33467; (OT101 Cycles) 4to11 = 0.09717; Dexamethasone use = -0.23876; (KPS score) 90 to 100 = 0.09747; Total Cumulative OT101 Dose = 0.00390; (Best Overall Response) responders = 2.88321; Log(scale) = -1.33100. Depicted are simulated PFS outcomes based on multivariate predictive modelling of PFS times for AA as well as GBM patients (Simulation parameters: Dexamethasone use: None or minimal; KPS score: 90-100; Number of OT101 cycles: 4-11 (viz: efficacy population), Cumulative OT101 dose: 46 mg/m^2 (=mean value for the mITT population); Age: 46 years (=mean age for mITT population) yielded 18-fold higher median PFS times for favorable responders vs. non-responders ($p < 0.00001$).

Table S1. Analysis Populations of Patients Randomized to Treatment with OT101.

**Treatment group**	**OT101 2.5mg/cycle N(AA/GBM)**	**OT101 19.8mg/cycle N(AA/GBM)**	**Combined Total N(AA/GBM)**
**Patient population**			
Intent-to-Treat (ITT)	48 (14/34)	50 (16/34)	98 (30/68)
Discontinued after randomization (before catheter-port surgery in OT101 groups)	7 (2/5)	1(1/0)	8 (3/5)
Safety population (Catheter surgery and/or OT101 treatment)	41 (12/29)	49 (15/34)	90 (27/63)
Discontinued after catheter surgery, before OT101 administration	1 (0/1)	0(0/0)	1 (0/1)
Modified Intent to Treat population (mITT)/Treated with OT101	40 (12/28)	49 (15/34)	89 (27/62)
Primary efficacy population (N=77)/ Treated with the intended minimum number of 4 cycles (Range: 4-11 cycles) of OT101	36 (12/24)	41 (14/27)	77 (26/51)

N: number of patients; AA: Anaplastic astrocytoma (WHO Grade 3); GBM: Glioblastoma multiforme (WHO Grade 4).

Table S2. Overview of Adverse Events in Safety Population.

**Patient category according to AE**	**OT101 2.5 mg/cycle (N=41)**		**OT101 19.8 mg/cycle (N=49)**		**Combined Total – Safety population (N=90)**	
**Entire study population**	**N (%)**	**n§**	**N (%)**	**n§**	**N (%)**	**n§**
Patients with AEs leading to discontinuation of treatment	19 (46.3)	57	24 (49.0)	78	43 (47.8)	135
Patients with Grade 3/4 AEs	31 (75.6)	162	37 (75.5)	161	68 (75.6)	323
Patients with AEs leading to death	11 (26.8)	11	17 (34.7)	17	28 (31.1)	28
Patients with drug related or possibly drug related AEs	11 (26.8)	28	21 (42.9)	53	32 (35.6)	81
Patients with SAEs	32 (78.0)	65	37 (75.5)	65	69 (76.7)	130
Patients with Procedure-related SAEs§§	14 (34.1)	21	14 (28.6)	16	28 (31.1)	37
Patients with drug related or possibly drug related SAEs	0 (0.0)	0	3 (6.1)	4	3 (3.3)	4
Patients with SAEs leading to permanent discontinuation of treatment	13 (31.7)	14	20 (40.8)	23	33 (36.7)	37

N: number of patients in the treatment group; n (%): number of patients with AEs (percent, based on N); n§: number of AEs; A patient may have findings in more than one category; §§Procedure-related SAEs were assessed by the investigator.

Table S3. Procedure-related Serious Adverse Events.

SAE according to MedDRA SOC, Preferred Term

	**OT101 2.5 mg/cycle (N=41)**		**OT101 19.8 mg/cycle (N=49)**		**Combined Total – Safety Population (N=90)**	
**Entire study population**	**N (%)**	**n§**	**N (%)**	**n§**	**N (%)**	**n§**
**Patients with at least one SAE**	14 (34.1)	21	14 (28.6)	16	28 (31.1)	37
**Infections and infestations**	3 (7.3)	5	3 (6.1)	4	6 (6.7)	9
Brain abscess	1 (2.4)	1	1 (2.0)	1	2 (2.2)	2
Central nervous system abscess	0 (0.0)	0	1 (2.0)	1	1 (1.1)	1
Meningitis	1 (2.4)	2	2 (4.1)	2	3 (3.3)	4
Pneumonia	1 (2.4)	1	0 (0.0)	0	1 (1.1)	1
Sepsis	1 (2.4)	1	0 (0.0)	0	1 (1.1)	1
**Injury, poisoning and procedural complications**	9 (22.0)	13	9 (18.4)	9	18 (20)	22
Accidental overdose	1 (2.4)	1	1 (2.0)	1	2 (2.2)	2
Application site abscess	0 0.0)	0	1 (2.0)	1	1 (1.1)	1
Application site infection	5 (12.2)	8	5 (10.2)	5	10 (11.1)	13
Application site inflammation	0 (0.0)	0	1 (2.0)	1	1 (1.1)	1
Incorrect route of drug administration	1 (2.4)	1	0 (0.0)	0	1 (1.1)	1
Medical device complication	3 (7.3)	3	1 (2.0)	1	4 (4.4)	4
**Nervous system disorders**	2 (4.9)	2	2 (4.1)	2	4 (4.4)	4
Brain edema	0 (0.0)	0	1 (2.0)	1	1 (1.1)	1
Cerebral hemorrhage	1 (2.4)	1	0 (0.0)	0	1 (1.1)	1
Cerebrospinal fistula	1 (2.4)	1	0 (0.0)	0	1 (1.1)	1
Hemiparesis	0 (0.0)	0	1 (2.0)	1	1 (1.1)	1
**Vascular disorders**	1 (2.4)	1	1 (2.0)	1	2 (2.2)	2
Deep vein thrombosis	0 (0.0)	0	1 (2.0)	1	1 (1.1)	1
Thrombophlebitis	1 (2.4)	1	0 (0.0)	0	1 (1.1)	1

Procedure-related SAEs Preventing the Start of or Leading to Discontinuation of OT101

**MedDRA SOC, Preferred Term**	**OT101 2.5 mg/cycle (N=41)**		**OT101 19.8 mg/cycle (N=49)**		**Combined Total – Safety Population (N=90)**	
**Entire Study population**	**N (%)**	**n§**	**N (%)**	**n§**	**N (%)**	**n§**
**Patients with at least one SAE**	6 (14.6)	6	6 (12.2)	7	12 (13.3)	13
**Infections and infestations**	2 (4.9)	2	3 (6.1)	4	5 (5.6)	6
Brain abscess	1 (2.4)	1	1 (2.0)	1	2 (2.2)	2
Central nervous system abscess	0 (0.0)	0	1 (2.0)	1	1 (1.1)	1
Meningitis	0 (0.0)	0	2 (4.1)	2	2 (2.2)	2
Sepsis	1 (2.4)	1	0 (0.0)	0	1 (1.1)	1
**Injury, poisoning and procedural complications**	3 (7.3)	3	3 (6.1)	3	6 (6.7)	6
Application site abscess	0 (0.0)	0	1 (2.0)	1	1 (1.1)	1
Application site infection	2 (4.9)	2	1 (2.0)	1	3 (3.3)	3
Application site inflammation	0 (0.0)	0	1 (2.0)	1	1 (1.1)	1
Incorrect route of drug administration	1 (2.4)	1	0 (0.0)	0	1 (1.1)	1
**Nervous system disorders**	1 (2.4)	1	0 (0.0)	0	1(1.1)	1
Cerebral hemorrhage	1 (2.4)	1	0 (0.0)	0	1(1.1)	1

N: number of patients in the treatment group; n (%): number of patients with AEs (percent, based on N); n§: number of AEs; A patient may have findings in more than one category.

Table S4. Display of Procedure-Related Serious Adverse Events.

Treatment	Dx	Pat. No.	SAE Term	Duration	Outcome	Comments
**OT101, 2.5 mg/cycle**	AA	102	Inadvertently applied 14 mL gadolinium and 10 mL NaCl intracerebral	103 days	Recovered with sequelae	OT101 permanently discontinued
	AA	105	Infection of port cavity	15 days	Recovered without sequelae	Therapy continued
	AA	302	Thrombophlebitis	Unknown (< 5 weeks)	Recovered without sequelae	Prophylactic surgery measure, therapy continued
	AA	403	Brain abscess left frontoparietal region	35 days	Recovered without sequelae	OT101 permanently discontinued
	AA	409	Ventricular catheter penetrated the ventricle	1 day	Recovered without sequelae	Relocation of catheter under local anesthesia before start of therapy
			Infection near the port chamber	19 days	Recovered without sequelae	Therapy continued
			Skin and subcutaneous tissue infection near port chamber	8 days	Recovered without sequelae	Therapy continued
	AA	417	Cellulitis (redness at port site)	17 days	Recovered without sequelae	Therapy interrupted
			Cellulitis (redness and pain chest wound site pump chamber)	8 days	Recovered without sequelae	Therapy continued
			Cellulitis (redness and pain chest wound site pump chamber)	5 days	Recovered without sequelae	Therapy continued
	AA	702	Accidental drug overdose due to pump handling error	1 day	Recovered without sequelae	Therapy continued
	GBM	124	Meningitis	17 days	Recovered without sequelae	Therapy continued
			Meningitis	8 days	Worsening	Leading to SAE sepsis, therapy permanently discontinued
			Sepsis	4 days	Death	
	GBM	141	Misplacement of medication catheter	11 days	Recovered without sequelae	Therapy started after SAE resolved

Treatment	Dx	Pat. No.	SAE Term	Duration	Outcome	Comments
	GBM	144	CSF Fistula along port catheter	21 days	Recovered without sequelae	Therapy continued with short interruptions
	GBM	320	Bleeding into right parieto-occipital area	45 days	Ongoing at time of death	Therapy was not started
			Pneumonia	43 days	Death	Therapy was not started
	GBM	323	Wound infection skin, left occipital	25 days	Recovered without sequelae	Removal of drug delivery system
	GBM	432	Postoperative wound infection	18 days	Recovered without sequelae	Removal of drug delivery system
	GBM	436	Misplacement of intratumoral catheter into the ventricle	1 day	Recovered without sequelae	Relocation of catheter before start of therapy
**OT101, 19.8 mg/cycle**	AA	201	Wound infection leading to removal of port system	19 days	Recovered without sequelae	OT101 permanently discontinued
	AA	301	Serous skin fistula at the catheter site	Unknown (< 8 weeks)	Recovered without sequelae	SAE after explantation of catheter
	AA	415	Right parietal abscess	49 days	Recovered with sequelae	OT101 permanently discontinued
			Meningitis	15 days	Recovered with sequelae	OT101 permanently discontinued
	GBM	127	Occlusion alarms due to short gripper needle	6 days	Recovered without sequelae	Therapy continued
			Deterioration of preexisting hemiparesis after surgery	41 days	Worsening	Therapy start delayed
	GBM	128	Abscess (intracerebral at catheter site)	18 days	Recovered without sequelae	Occurred after end of therapy
	GBM	134	Infection of catheter/port system	7 days	Recovered without sequelae	SAE occurred 1 months after end of therapy
	GBM	139	Subcutaneous catheter infection, left temporal region	41 days	Recovered without sequelae	Therapy continued
	GBM	143	Inflammation of the wound	36 days	Recovered without sequelae	OT101 permanently discontinued 4 weeks after start of SAE
	GBM	149	Brain abscess	113 days	Recovered without sequelae	OT101 permanently discontinued
	GBM	220	Meningitis	46 days	Recovered with sequelae	OT101 permanently discontinued

Treatment	Dx	Pat. No.	SAE Term	Duration	Outcome	Comments
	GBM	322	Increase of preexisting intracerebral edema	45 days	Ongoing at time of death	Therapy continued
	GBM	435	Catheter site cellulitis	19 days	Recovered without sequelae	Therapy continued
	GBM	442	Left lower limb deep venous thrombosis (Grade 3)	21 days	Recovered without sequelae	Therapy continued
	GBM	444	Accidental drug overdose due to pump handling error	1 day	Recovered without sequelae	Therapy continued

Table S5. Incidence of OT101-Related or Possibly related Toxicity Grade 3 or 4 AE.

	OT101 2.5 mg/cycle (N=41)		OT101 19.8 mg/cycle (N=49)		Combined total – Safety population (N=90)	
**Study population**	**N (%)**	**n§**	**N (%)**	**n§**	**N (%)**	**n§**
**Patients with at least one AE**	2 (4.9)	3	8 (16.3)	13	10 (11.1)	16
**Infections and infestations**	0 (0.0)	0	1 (2.0)	1	1 (1.1)	1
Meningitis	0 (0.0)	0	1 (2.0)	1	1 (1.1)	1
**Injury, poisoning and procedural complications**	0 (0.0)	0	1 (2.0)	1	1 (1.1)	1
Application site reaction	0 (0.0)	0	1 (2.0)	1	1 (1.1)	1
**Metabolism and nutrition disorders**	0 (0.0)	0	2 (4.1)	2	2 (2.2)	2
Hyponatremia	0 (0.0)	0	2 (4.1)	2	2 (2.2)	2
**Nervous system disorders**	2 (4.9)	3	4 (8.2)	7	6 (6.7)	10
Brain edema	0 (0.0)	0	2 (4.1)	2	2 (2.2)	2
Aphasia	1 (2.4)	1	0 (0.0)	0	1 (1.1)	1
Dizziness	0 (0.0)	0	1 (2.0)	1	1 (1.1)	1
Hemiparesis	0 (0.0)	0	2 (4.1)	2	2 (2.2)	2
Monoparesis	1 (2.4)	1	0 (0.0)	0	1 (1.1)	1
Facial paresis	0 (0.0)	0	1 (2.4)	1	1 (1.1)	1
Sensory disturbance	1 (2.4)	1	0 (0.0)	0	1 (1.1)	1
Simple partial seizures	0 (0.0)	0	1 (2.0)	1	1 (1.1)	1
**Psychiatric disorders**	0 (0.0)	0	1 (2.0)	2	1 (1.1)	2
Euphoric mood	0 (0.0)	0	1 (2.0)	1	1 (1.1)	1
Self-esteem decreased	0 (0.0)	0	1 (2.0)	1	1 (1.1)	1

N: number of patients in the treatment group; n (%): number of patients with AEs (percent, based on N); n§: number of AEs; A patient may have findings in more than one category.

Table S6. OT101-Related or Possibly OT101-Related Adverse Events and Serious Adverse Events Leading to Discontinuation of OT101.

OT101-Related or Possibly OT101-Related AE Leading to Discontinuation of OT101

UPN	Tumor Type	Treatment Group mg/cycle	Toxicity Grade	SAE Yes/No	AE (Preferred Term)
**0104**	AA	OT101, 19.8	Grade 2	No	*Brain edema*
**0422**	GBM	OT101, 19.8	Grade 3	Yes	*Hyponatremia*
			Grade 2	Yes	*Brain edema*
**0538**	GBM	OT101, 19.8	Grade 2	Yes	*Cerebral disorder*
**0319**	GBM	OT101, 19.8	Grade 3	No	*Brain edema*
**0421**	GBM	OT101, 19.8	Grade 3	No	*Brain edema*
**0147**	GBM	OT101, 19.8	Grade 2	No	*Brain compression*
**0220**	GBM	OT101, 19.8	Grade 4	No	*Application site reaction*
**0519**	GBM	OT101, 19.8	Grade 2	No	*Neutrophilia*
			Grade 2	No	*Leukocytosis*
**0535**	GBM	OT101, 19.8	Grade 4	No	*Hemiparesis*
			Grade 1	No	*Karnofsky Scale worsened*
**0524**	GBM	OT101, 2.5	Grade 2	No	*Arthralgia*
			Grade 2	No	*Asthenia*
			Grade 3	No	*Monoparesis*
			Grade 3	No	*Sensory disturbance*

OT101-Related or Possibly OT101-Related SAE Leading to Discontinuation of OT101 in Safety Population

UPN	Tumor Type	Treatment group (mg/cycle)	Toxicity Grade	SAE (Preferred Term)	Procedure-related	Action taken
0422	GBM	OT101, 19.8	Grade 3	Hyponatremia	No	Study drug permanently discontinued;
			Grade 2	Brain edema	No	Study drug permanently discontinued
0538	GBM	OT101, 19.8	Grade 2	Cerebral disorder	No	Study drug permanently discontinued

Note: NCI-CTC Toxicity Grade: 0 = none, 1 = mild, 2 = moderate, 3 = severe, 4 = life-threatening.

Table S7. Incidence of OT101-Related or - Possibly Related Adverse Events Causing Discontinuation of OT101.

	OT101 2.5 mg/cycle (N=41)		OT101 19.8 mg/cycle (N=49)		Combined Total – Safety Population (N=90)	
**Study population**	**N (%)**	**n§**	**N (%)**	**n§**	**N (%)**	**n§**
**Patients with at least one AE**	1 (2.4)	4	9 (18.4)	13	10 (11.1)	17
**Blood and lymphatic disorders**	0 (0.0)	0	1 (2.0)	2	1 (1.1)	2
Leukocytosis	0 (0.0)	0	1 (2.0)	1	1 (1.1)	1
Neutrophilia	0 (0.0)	0	1 (2.0)	1	1 (1.1)	1
**General disorders and administration site conditions**	1 (2.4)	1	0 (0.0)	0	1 (1.1)	1
Asthenia	1 (2.4)	1	0 (0.0)	0	1(1.1)	1
**Infections and infestations**	0 (0.0)	0	1 (2.0)	1	1 (1.1)	1
Meningitis	0 (0.0)	0	1 (2.0)	1	1 (1.1)	1
**Injury, poisoning and procedural complications**	0 (0.0)	0	1 (2.0)	1	1 (1.1)	1
Application site reaction	0 (0.0)	0	1 (2.0)	1	1 (1.1)	1
**Investigations**	0 (0.0)	0	1 (2.0)	1	1 (1.1)	1
Karnofsky scale worsened	0 (0.0)	0	1 (2.0)	1	1 (1.1)	1
**Metabolism and nutrition disorders**	0 (0.0)	0	1 (2.0)	1	1 (1.1)	1
Hyponatremia	0 (0.0)	0	1 (2.0)	1	1 (1.1)	1
**Musculoskeletal and connective tissue**	1 (2.4)	1	0 (0.0)	0	1 (1.1)	1
Arthralgia	1 (2.4)	1	0 (0.0)	0	1 (1.1)	1
**Nervous system disorders**	1 (2.4)	1	7 (14.3)	7	8 (8.9)	8
Brain compression	0 (0.0)	0	1 (2.0)	1	1 (1.1)	1
Brain edema	0 (0.0)	0	4 (8.2)	4	4 (4.4)	4
Cerebral disorders	0 (0.0)	0	1 (2.0)	1	1 (1.1)	1
Hemiparesis	0 (0.0)	0	1 (2.0)	1	1 (1.1)	1
Monoparesis	1 (2.4)	1	0 (0.0)	0	1 (1.1)	1
Sensory disturbance	1 (2.4)	1	0 (0.0)	0	1 (1.1)	1

N: number of patients in the treatment group; n (%): number of patients with AEs (percent, based on N); n§: number of AEs; A patient may have findings in more than one category.

Table S8. Baseline Patient Characteristics for the Modified Intent-to-Treat and Efficacy Populations.

Parameter	Modified Intent to Treat (mITT) Population (N=89)	Efficacy Population (N=77)
**Diagnosis – N (%)**		
AA (WHO grade III)	27 (30.3)	26 (33.8)
GBM (WHO grade IV)	62 (69.7)	51 (66.2)
**Gender – N (%)**		
Female	25 (28.1)	21 (27.3)
Male	64 (71.9)	56 (72.7)
**Race – N (%)**		
Caucasian	58 (65.2)	49 (63.6)
Asian	31 (34.8)	28 (34.6)
Black	0 (0.0)	0 (0.0)
**OT101 Dose Cohort – N (%)**		
Low (2.5 mg/cycle)	40 (44.9)	36 (46.8)
High (19.8 mg/cycle)	49 (55.1)	41 (53.2)
**OT101 Exposure**		
**# OT101 Cycles**: Median (Range)	6 (1-11)	7 (4-11)
Mean±SE	7±0.3	7.8±0.3
**Total OT101 Dose (mg/m^2)**: Median(Range)	22.7 (1.1 - 152.1)	41.7 (4.7-152.1)
Mean±SE	45.2 ± 4.6	49.6±5.1
**Age (Years)**		
Median (Range)	45 (19-73)	44 (19-73)
Mean ± SE	46.3±1.3	45.5±1.4
**KPS at Randomization**		
Median (Range)	90 (70-100)	90 (70-100)
Mean ± SE	88 ±1	88±1
**Size of Target Tumor Lesion**		
2-D in cm^2 - Median (Mean ± SE)	8.6 (9.3±0.5)	8.5 (8.4±0.7)
3-D in cm^3 - Median (Mean ± SE)	21.5 (27.2±2.5)	21.2 (21.3±2.2)

Table S9. Single Agent Clinical Activity of Intratumorally Administered OT101 in R/R High Grade Glioma Patients.

				Target Lesion		Best Overall Response							
**Patient ID**	**Diagnosis**	**1st Line Therapy**	**# of OT101 Cycles (dose level–mg/cycle)**	**2-D (cm²)**	**3-D (cm³)**	**McDonald Criteria**	**Modified McDonald Criteria**	**Time to PR (days)**	**Time to CR (days)**	**Duration of CR/PR (days)**	**PFS (days)**	**OS (days)**	**Survival Status at last follow-up**
309538	GBM	Resection, RAD(64Gy)	4(19.8)	13.1±0.8	33.9±9.0	PR	PR	742	NA	≥114	≥856	≥1663	A
301525	GBM	Resection,RAD(60Gy), Chemo(VCR+CCNUx3)	11(2.5)	10.9±0.3	30.8±0.7	CR	CR	282	1120	≥1562	≥1844	≥1844	A
304532	GBM	Resection,RAD(60 Gy)	4(19.8)	3.7±1.2	5.9±2.5	PR	PR	133	NA	184	374	855	D
108138	GBM	Resection,RAD(60 Gy), Chemo (us)[#]	11(2.5)	14.7±1.3	44.5±2.5	PR	PR	321	NA	57	≥378	785	D
306539	AA	RAD (us); Resection	7(2.5)	9.9±0.4	22.3±1.3	PR	PR	37	NA	28	≥94	1280	D
310510	AA	RAD (us)	11(19.8)	11.2*	21.3*	PR	PR	234	NA	≥1383	≥1617	≥1617	A
404702	AA	Resection,RAD(60Gy), Chemo(Paclitaxelx6)	11(2.5)	6.1±0.5	14.6±4.6	PR	PR	237	NA	1100	1281	1492	D
404705	AA	Resection,RAD(60 Gy), Chemo(TMZx1)	11(2.5)	6.9±1.1	16.3±3.9	PR	PR	915	NA	400	1423	≥1467	A
403408	AA	RAD(59.4 Gy)	11(19.8)	7.9±0.8	16.9±4.2	PR	PR	350	NA	109	994	1072	D
405402	AA	Resection,RAD(45 Gy)	11(19.8)	1.8±0.2	1.9±0.2	PR	PR	452	NA	176	628	≥1902	A
405704	AA	Resection,RAD(60 Gy)	11(19.8)	4.8±2.0	9.2±4.7	CR	CR	119	917	≥1350	≥1469	≥1544	A
203302	AA	Resection, RAD (60 Gy), Chemo (PCVx5)	10(2.5)	9.3±0.3	21.1±1.3	CR	CR	483	1838	≥1516	≥2000	≥2000	A
305503	AA	Resection,RAD(60Gy), Chemo (HU)	11(2.5)	4.2±0.9	8.5±2.3	PR	PR	430	NA	57	1109	1243	D
404417	AA	Resection,RAD(60Gy), Chemo(Paclitaxel x6)	11(2.5)	8.6±0.7	25.8±2.1	PR	PR	185	NA	224	1070	1136	D
403405	AA	RAD(59.4 Gy)	11(19.8)	6.5±0.4	16.7±2.9	PR	PR	289	NA	283	572	1172	D
405412	GBM	Resection,RAD(80 Gy)	11(2.5)	3.8±1.2	4.6±1.7	PR	PR	307	NA	126	899	1069	D
405413	AA	Resection,RAD(60 Gy)	11(2.5)	8.6±1.4	23.8±4.5	PR	PR	287	NA	133	420	811	D
407409	AA	Resection,RAD(55 Gy),	11(2.5)	8.4±0.3	24.9±3.3	PR	PR	231	NA	219	450	963	D
502201	AA	Resection,RAD(60 Gy)	8(19.8)	13.4±3.1	41.5±16.9	PR	PR	260	NA	71	331	1079	D
107102	AA	RAD (60Gy),Chemo (ACNU/VM26 x 4)	7(2.5)	12.8±0.5	41.8±1.0	SD	SD	NA	NA	NA	1278	1743	D
301524	GBM	Resection,RAD (60Gy), Chemo (Lomustin+VCR x 2)	10(2.5)	10.8±2.0	24.7±5.1	SD	SD	NA	NA	NA	1135	1520	D
102141	GBM	Resection, RAD, Chemo (ACNU x 4)	11(2.5)	7.6±2.8	17.0±7.3	SD	SD	NA	NA	NA	185	702	A
102149	GBM	Resection, RAD (60 Gy),Chemo (TMZ x 3)	10(19.8)	4.2±0.9	11.5±7.3	SD	SD	NA	NA	NA	643	1116	D
404406	AA	Resection, RAD (60Gy), Chemo(Carbo x 6)	11(2.5)	7.8±0.8	19.7±3.4	SD	SD	NA	NA	NA	295	1797	A
404435	GBM	Resection, RAD (60Gy) Chemo (TMZ)	11(19.8)	12.2±0.3	33.6±2.5	SD	SD	NA	NA	NA	414	648	D
306545	GBM	Resection, RAD (70Gy)	11(2.5)	9.2±1.5	21.6±5.4	SD	SD	NA	NA	NA	767	1480	A

In **203302**, a patient with pseudo-progression early in the course who had subsequently achieved a PR, no residual measurable or non-measurable lesions were detected in the follow-up MRI done on day 1839 and a confirmatory MRI done > 4 weeks later on day 2000 and the response was formally identified by the central review as CR on day 2000. Patient was off steroids throughout his course on this study. In patient, 405412 another patients with early pseudo-progression who likewise had a PR, the response also deepened, and no residual lesions were detected on the MRI done on day 432, but the BOR did not qualify as CR due to lack of a confirmatory follow-up MRI to document at least 4-weeks of duration of the deep response. Patient **306539** had an early response to OT101 with >50% reduction of the target lesion on day 37 MRI (Baseline: 22,271 mm^3; Lesion size on Day 37: 5,985 mm^3 [73.1% reduction from baseline] based on the independent reads of 2 central reviewers) and this short-lived response was confirmed as PR by central review on day 64 (Lesion size: 6,824 mm^3; 69.3% reduction from baseline). Patient **309538** had a significant shrinkage of the treated target lesion starting on day 248 after randomization. The MRI scan done 742 days after randomization showed a shrinkage of the target lesion to a size of 8,073 mm^3 from the baseline size of 33,911 mm^3 (76.2% reduction from baseline) based on the independent reads of 3 central reviewers. The follow-up MRI on day 855 post randomization showed continued shrinkage based on the independent reads from 3 central reviewers and the lesion size was down to 5,350 mm^3 (84.2% reduction from baseline) and this response was confirmed as PR by central review. In patient **310510**, the BOR was based on local review of the MRI data; the BOR determinations in other patients were based on central review. In **404705**, the PD was an unconfirmed single point determination based on a follow-up MRI. us: unspecified; A: alive; D: dead; SD: stable disease; CR: complete response; PR: partial response.

Table S10. Grade III/IV AE and SAE in R/R High Grade Glioma Patients Showing an Objective Response or a Prolonged Stable Disease to Intratumorally Administered OT101.

Best Overall Response = CR or PR

Patient ID	Reported Term (Grade)	SAE (Yes/No)	Causality	Onset - End	Modification of therapy	Outcome
309538	Right hemiparesis (2)	Yes	Possibly related	D64-D81	Yes*	Recovered (F)
301525	Brain edema (4)	Yes	Unlikely	D6-D76	No	Recovered (F)
	Hemorrhage (4) - brain tumor	No	Unlikely	D6-D29	No	Recovered (F)
	Head injury (3)		Not related	D6-D15	No	Recovered (F)
	Peripheral sensory neuropathy (3)	No	Unlikely	D6-D29	No	Recovered (F)
	Peripheral motor neuropathy (3)	No	Unlikely	D75-D85	No	Recovered (F)
304532	None	NA	NA	NA	NA	NA
108138	None	NA	NA	NA	NA	NA
306539	Seizures (3)	No	Not related	D86	No	Recovered (F)
	Depression (3)	No	Not related	D86-D92	No	Recovered (F)
310510	None	NA	NA	NA	NA	NA
404702	None	NA	NA	NA	NA	NA
404705	None	NA	NA	NA	NA	NA
403408	Partial seizures (3)	Yes	Unlikely	D93-D95	No	Recovered (F)
405402	Vomiting/Gastroenteritis (3)	Yes	Unlikely	D44-D46	No	Recovered (F)
405704	None	NA	NA	NA	NA	NA
203302	Generalized tonic-clonic seizure (3)	No	Not related	D149	No	Recovered (F)
305503	None	NA	NA	NA	NA	NA
404417	Catheter site cellulitis (1)	Yes	Unlikely	D84; D120-D127; D152-156	Yes (Cycle interval increased)	Recovered (F)
403405	None	NA	NA	NA	NA	NA
405412	None	NA	NA	NA	NA	NA
405413	Pregnancy (4)	Yes	Not related	D10-D17	No	Recovered (F)
407409	Catheter complication (2)	Yes	Not related	D1	No	Recovered (F)
	Leukopenia (2)	Yes	Unlikely	D28-D31	No	Recovered (F)
	Leukopenia (1)	Yes	Unlikely	D91-D94	No	Recovered (F)
502201	Seizure (1)	Yes	Not related	D5-D16	No	Recovered (F)
	Surgical scar infection (2)	Yes	Not related	D100-D118	Yes*	Recovered (F)
	Tumor progression (4)	Yes	Not related	D770	No	Recovered (F)

Best Overall Response = SD>6 months

Patient ID	Reported Term (Grade)	SAE (Yes/No)	Causality	Onset - End	Modification of therapy	Outcome
1070102	Low back pain (3)	Yes	Unlikely	D101-NA	Yes*	Ongoing at EOS
	Inadvertent application of CM (4)	Yes	Not related	D93-D196	Yes*	Recovered (F)
	Confusion due to high ICP after	No	Not related	D93-D98	Yes*	Recovered (F)

	inadvertent application of CM (3)					
	Aphasia (4)	No	Not related	D103-D121	Yes*	Recovered (I)
	Ataxia (3)	No	Unlikely	D93-NA	Yes*	Ongoing at EOS
	Aphasia (3)	No	Not related	D110-NA	No	Ongoing at EOS
3010524	Hemiparesis, left (3)	No	Possibly related	D151-NA	Yes*	Ongoing at EOS
	Sensitivity disorder (3)	No	Possibly related	D151-NA	Yes*	Ongoing at EOS
1020141	Hemiparesis, right (3)	No	Unlikely	D220-NA	No	Ongoing at EOS
	Misplacement of catheter (3)	Yes	Not related	D1-D12	No	Recovered (F)
	Aphasia (3)	Yes	Unlikely	D220-NA	No	Ongoing at EOS
1020149	Leukocytopenia (3)	No	Not related	D13-D27	No	Recovered (F)
	Brain abscess (3)	Yes	Unlikely	D145-D153	Yes*	Recovered (F)
4040406	None	NA	NA	NA	NA	NA
4040435	Hemiparesis (4)	No	Not related	D485-NA	No	Ongoing at EOS
3060545	None	NA	NA	NA	NA	NA

D: Day; *Permanently discontinued; F: fully, without sequelae; I: incomplete, with sequelae; EOS: end of study.

Table S11. GLM-based multivariate analysis to further evaluate the predictive value of clinical parameters for favorable clinical responses to OT101 therapy.

	Efficacy Population		mITT Population	
**Predictor**	χ^2	**P-value**	χ^2	**P-value**
Age	0.0	1.0	0.0	1.0
KPS Score	6.4	0.011	5.8	0.016
Diagnosis (AA vs GBM)	5.7	0.017	6.4	0.012
Total OT101 Dose	3.7	0.055	2.3	0.126
Dexamethasone Use	14.2	0.007	16.6	0.002
Size of Target Lesion	0.0	1.0	0.0	1.0
Previous Therapy	3.6	0.3	2.6	0.5

Table S12. Multivariate Analysis of Progression-Free Survival and Overall Survival, According to Risk Factor.

Risk Factor	PFS (Days) Median (95% CI)	Z-stat -BOR/ +BOR	P-Value -BOR/ +BOR	OS (Days) Median (95% CI)	Z-stat -BOR/ +BOR	P-Value -BOR/ +BOR
**Diagnosis**						
GBM (N=62)	38 (35-61)	3.9 / 2.4	0.0001/ 0.018	274 (180-399)	3.9 / 2.0	0.0001 / 0.04
AA (N=27)	994 (118-1423)			1136 (811-1743)		
**Age (Years)**						
53-73 (N=30)	36 (35-86)	0.2 / 0.5	0.8 / 0.6	213 (137-341)	0.6 / 0.3	0.6 / 0.7
19-41 (N=30)	101(59-1109)			803 (365-1243)		
**KPS Score**						
70-80 (N=25)	40 (36-67)	2.6 / 0.8	0.009 / 0.4	162 (131-341)	3.3 / 2.1	0.0009 / 0.03
90-100 (N=64)	88 (40-295)			445 (399-1069)		
**OT101 Cycles**						
1-3 (N=12)	32 (32-NA)	1.3 / 0.6	0.2 / 0.5	128 (93-NA)	2.7 / 2.1	0.007 / 0.03
4-11 (N=77)	86 (40-134)			432 (299-788)		
**Total OT101 Dose (mg/m^2)**						
1.1-14 (N=30)	36 (35-60)	0.4 / 2.7	0.7 / 0.006	222 (152-406)	1.2 / 2.3	0.2 / 0.02
53-152 (N=30)	88 (40-176)			447 (402-1079)		
**Dexamethasone Use**						
Extensive (N=25)	36 (35-86)	5.4 / 1.7	<0.00001 / 0.08	273 (152-432)	5.1 / 2.3	<0.00001 / 0.02
No or Minimal (N=28)	643 (295-NA)			1172(963-NA)		
**Best Overall Response (BOR)**						
Non-responder - SD<6 months or PD (N=63)	36 (35-54)	- / 16.5	- / <0.00001	229 (163-318)	- / 5.8	- / <0.00001
Favorable responder - PR, CR or SD>6 months (N=26)	1109 (992-NA)			1280 (1116-NA)		

© 2019 by the authors. Licensee MDPI, Basel, Switzerland. This article is an open access article distributed under the terms and conditions of the Creative Commons Attribution (CC BY) license (http://creativecommons.org/licenses/by/4.0/).
